# A human iPSC-derived hepatocyte screen identifies compounds that inhibit production of Apolipoprotein B

**DOI:** 10.1038/s42003-023-04739-9

**Published:** 2023-04-24

**Authors:** Jui-Tung Liu, Caren Doueiry, Yu-lin Jiang, Josef Blaszkiewicz, Mary Paige Lamprecht, James A. Heslop, Yuri K. Peterson, Juliana Debrito Carten, Paula Traktman, Yang Yuan, Salman R. Khetani, Waleed O. Twal, Stephen A. Duncan

**Affiliations:** 1 Department of Regenerative Medicine and Cell Biology, Medical University of South Carolina, Charleston, SC 29425 USA; 2 Drug Discovery and Biomedical Sciences, Medical University of South Carolina, Charleston, SC 29425 USA; 3 Biochemistry and Molecular Biology, Medical University of South Carolina, Charleston, SC 29425 USA; 4 Department of Biomedical Engineering, University of Illinois at Chicago, Chicago, IL 60607 USA; 5 Grùthan Biosciences LLC, Hollywood, SC 29449 USA

**Keywords:** Drug discovery, Cell biology

## Abstract

Familial hypercholesterolemia (FH) patients suffer from excessively high levels of Low Density Lipoprotein Cholesterol (LDL-C), which can cause severe cardiovascular disease. Statins, bile acid sequestrants, PCSK9 inhibitors, and cholesterol absorption inhibitors are all inefficient at treating FH patients with homozygous *LDLR* gene mutations (hoFH). Drugs approved for hoFH treatment control lipoprotein production by regulating steady-state Apolipoprotein B (apoB) levels. Unfortunately, these drugs have side effects including accumulation of liver triglycerides, hepatic steatosis, and elevated liver enzyme levels. To identify safer compounds, we used an iPSC-derived hepatocyte platform to screen a structurally representative set of 10,000 small molecules from a proprietary library of 130,000 compounds. The screen revealed molecules that could reduce the secretion of apoB from cultured hepatocytes and from humanized livers in mice. These small molecules are highly effective, do not cause abnormal lipid accumulation, and share a chemical structure that is distinct from any known cholesterol lowering drug.

## Abstract (web summary)

iPSC-derived hepatocytes are used to identify small molecule inhibitors of apoB secretion by the liver without causing steatosis.

## Introduction

Cardiovascular diseases are a common cause of mortality being responsible for an estimated 17.8 million deaths in 2017^1^. The cost of treating heart disease and stroke is expected to be greater than one trillion dollars by the year 2035^2^.

Familial hypercholesterolemia (FH) is an inherited disease that increases the risk of developing cardiovascular disease and is characterized by elevated plasma low-density lipoprotein-cholesterol (LDL-C) levels. The prevalence of heterozygous FH (HeFH) is between 1/200 and 1/500 people and of homozygous FH (hoFH) between 1/160,000 and 1/300,000^3^. FH is primarily caused by mutations in the low-density lipoprotein receptor (LDLR). The LDLR is an integral membrane protein that binds LDL particles from the circulation and mediates endocytosis^4,5^. LDL-cholesterol is cleared from the serum predominantly through LDLR uptake and catabolism by hepatocytes.

Statins are the primary treatment for hypercholesterolemia. They act by inhibiting HMG-CoA reductase, reducing cholesterol synthesis, and increasing LDLR expression in hepatocytes which leads to enhanced LDL-C clearance^6^. Although statins are effective in most populations, more than 20% of individuals show an inadequate response to statin treatment^7–9^, and two thirds of coronary artery disease patients fail to maintain cholesterol goals when using a statin alone^10^. Unfortunately, because statins act primarily by increasing expression of the LDLR, most hoFH patients are resistant to statin treatment. Recently, two antibodies to PCSK9, alirocumab and evolocumab, have been approved by the FDA for treatment of hypercholesterolemia. Like statins these treatments increase the steady-state level of the LDLR, promoting hepatic uptake of LDL^11^. Unfortunately, the use of PCSK9 inhibitors is expensive, limiting their widespread use and, depending on the specific mutations, can be ineffective against hoFH^11^.

Alternative drugs have recently become available to treat hoFH by controlling the production of LDL and its precursor VLDL by the hepatocytes. Lomitapide acts by inhibiting microsomal triglyceride transfer protein (MTP, MTTP). Inhibition prevents co-translational loading of triglyceride onto Apolipoprotein B (apoB), which is the central protein component of VLDL, and results in its proteolytic degradation^6^. Mipomersen is an antisense oligonucleotide that specifically binds to *APOB* mRNA to block protein translation^12^. Both Lomitapide and Mipomersen showed a substantial reduction of serum LDL and were approved to treat hoFH patients. Despite their effectiveness in lowering VLDL production, both treatments can increase hepatic fat and transaminase and can cause severe liver damage^13^. Therefore, there is a need to develop pharmaceuticals to lower VLDL in a broad population of patients including those with hoFH.

Attempts to develop small molecules to treat metabolic liver diseases have been hampered by the lack of an efficacious and reproducible in vitro model. Hepatoma cells do not accurately recapitulate hepatic disease phenotypes due to their tumorigenic origin^14^. Although primary hepatocytes show promising physiological characteristics, their limited availability, lot-to-lot variability, and difficulty in maintaining phenotypic function under simple culture conditions, reduce their suitability for high throughput screening^15^. Human iPSC-derived hepatocyte-like cells (iPSC-hepatocytes) have rapidly transformed the study of liver development and disease^16,17^. The capacity to produce large numbers of human hepatocyte-like cells derived from patients, allows investigators to perform high-throughput drug screens to discover therapies for monogenic liver diseases^18–20^.

In an effort to identify compounds for the potential treatment of hypercholesterolemia, we performed a high throughput screen using human iPSC-hepatocytes. We screened the South Carolina Compound Collection (SC^3^), a proprietary small molecule library that contains 130,000 fully annotated drug-like molecules. Compounds that reduced apoB in the culture medium were further tested in hoFH patient-derived iPSC-hepatocytes to identify candidates that act independently of the LDLR pathway. In contrast to MTP inhibitors, lead compounds had no effect on intracellular lipid accumulation and did not impact steady-state intracellular apoB protein levels. The compounds were not only effective in cultured cells, but also lowered serum cholesterol, triglyceride, apoB and Lp(a) levels in liver-humanized mice. Through this study we have identified a class of molecules that have potential to provide a treatment for patients with FH or primary hypercholesterolemia that is unresponsive to conventional therapeutics.

## Results

### Small molecule screen to identify reduced apoB production by human iPSC-derived hepatocyte-like cells

To identify compounds that reduce apoB-containing lipoproteins such as VLDL by hepatocytes, we used the stepwise differentiation of human iPSCs to hepatocyte-like cells (iPSC-hepatocytes)^21,22^. iPSC-hepatocytes recapitulate many aspects of human liver function, including the robust and stable secretion of VLDL^19,23^. ApoB accumulation in the culture medium, provides a reliable biomarker for the identification of compounds with the potential to reduce the production of VLDL by the liver because apoB is the central protein component of all VLDL particles^24^. We have previously validated an Enzyme-linked immunosorbent assays (ELISA)-based approach for identifying compounds that reduce secreted apoB (Z′ = 0.73)^25^. Therefore, we measured the level of apoB in the culture medium as the primary assay.

**Fig. 1:**
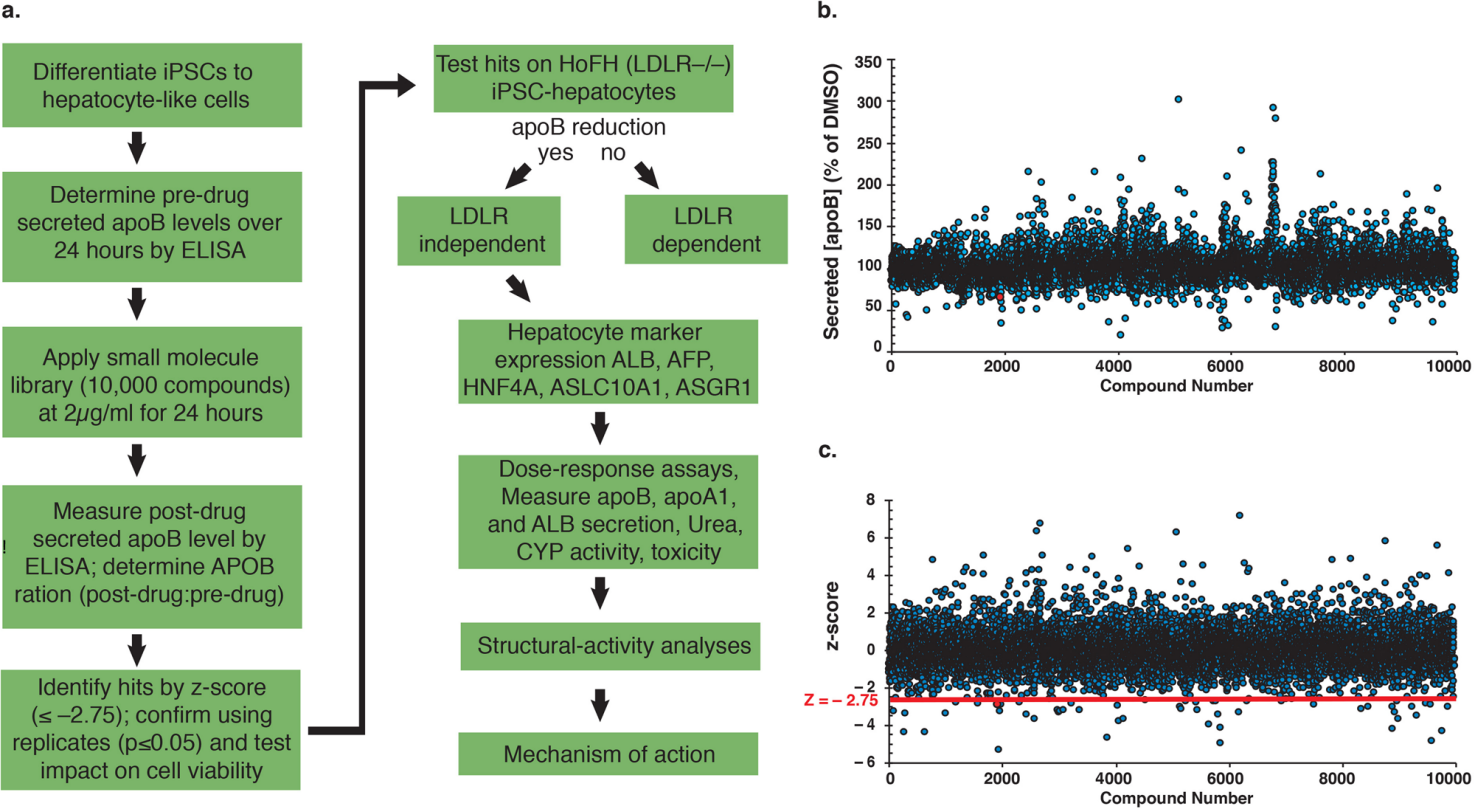
A small molecule screen using human iPSC-derived hepatocytes. **a** Schematic overview of the procedure used to identify compounds that reduce apoB produced by human iPSC-hepatocytes. **b** Graph showing post-drug: pre-drug ratios (∆ [apoB]) in the medium of apoB in compound-treated compared to DMSO-treated iPSC-hepatocytes (% DMSO). The blue dots represent each individual compound from the SC^3^ library 10 K representative set. **c** Graph showing the *z* score of each individual compound (blue dots) on the post-drug: pre-drug apoB ratio. Red dot is DL-1. Red line indicates the z-score at a value of –2.75 (*z* = –2.75).

Human iPSCs were differentiated to hepatocyte-like cells in a 96-well monolayer format. The level of apoB was measured by ELISA before and after treatment with 10,000 small molecules from the SC^3^ representative set (Fig. 1a). The SC^3^ library contains ~130,000 proprietary drug-like molecules with an average MW of 400 g/mol. The initial screening dose was 2 µg/ml (average 5 µM), which is within the range of the established acceptable dose of a high throughput screen^26^. The post-drug: pre-drug ratio of apoB in the culture medium was determined for each compound and normalized with the DMSO-treated group (Fig. 1b). The identification of hits was then determined by *z*-score analysis (Fig. 1c)^27^. Small molecules with a *z* score of ≤–2.75 were identified as primary hits. Using these parameters, we identified 46 compounds (SC3-01 through SC3-46) with an apoB ratio ranging from 0.82 to 0.2.

**Fig. 2:**
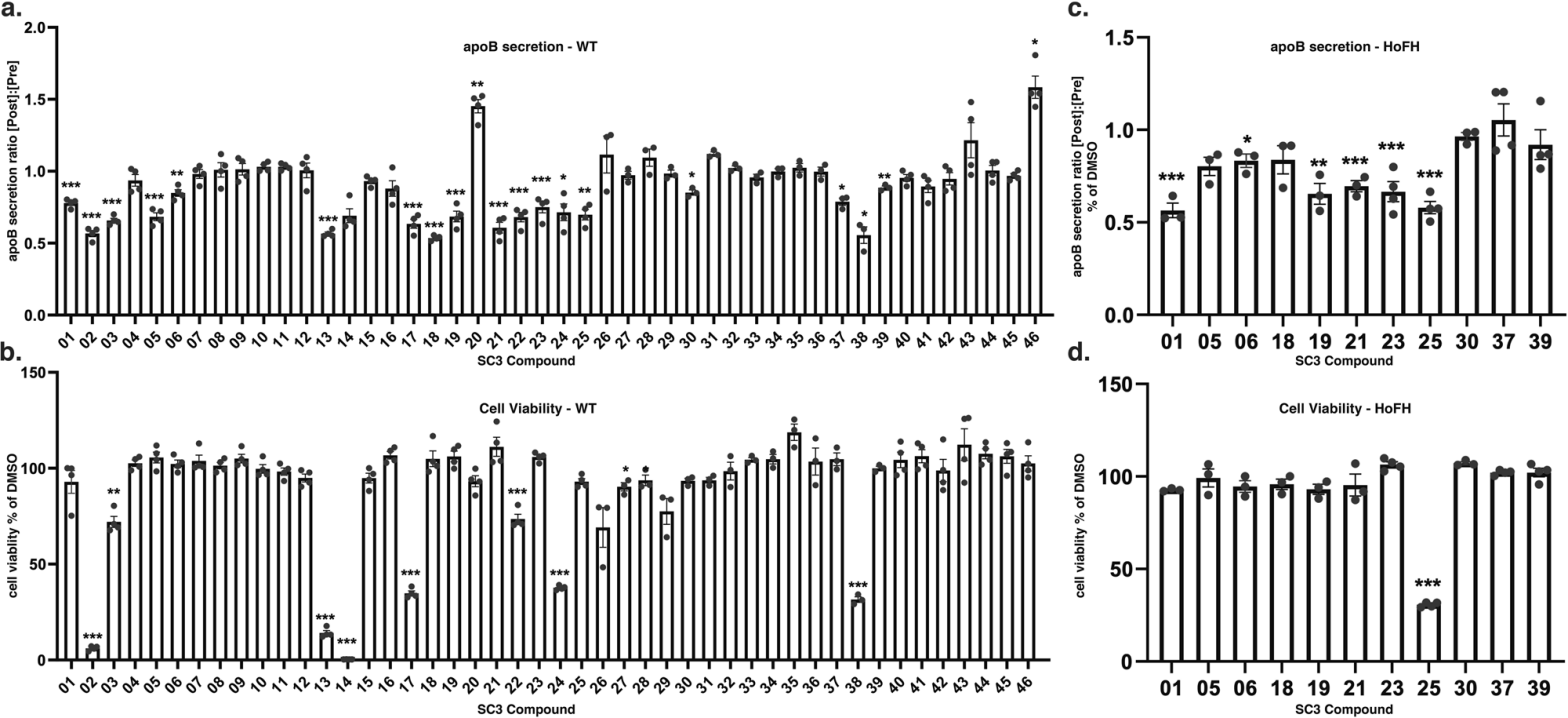
Confirmation of primary hits in normal donor and hoFH patient-derived iPSC-hepatocytes. **a**, **b** Forty-six hits (SC3-01 through SC3-46) from the initial screen were tested in human iPSC-derived hepatocytes at a concentration of 2 µg/mL. **a** ApoB levels in the medium were determined by sandwich ELISA assay. Bar graph shows the effect on accumulation of apoB over 24 h in the culture medium determined by comparing the post-drug and pre-drug apoB ratio normalized to DMSO-treated controls. **b** Cell viability was determined by ATP-based luminescence assay. **c**, **d** Compounds with a significant reduction in apoB without affecting cell viability were further tested in hoFH patient-derived iPSC-hepatocytes. **c** Bar graph showing the ratio of apoB in post-drug and pre-drug culture medium relative to DMSO controls. **d** Graph shows average cell viability relative to controls. All data are shown as mean ± SD of four replicates (*n* = 4). Each individual compound was compared to vehicle DMSO only. Statistical analyses were performed using Student’s *t* test; *p ≤ 0.05, *p* ≤ 0.01, ***p ≤ 0.001.

Compounds identified in the initial screen were subjected to a series of secondary assays (Fig. 2). First, we confirmed the reproducibility of each compound to lower apoB in the medium of wild type iPSC-hepatocytes and discarded any compound that failed to show a significant and reproducible reduction in apoB compared to vehicle (Fig. 2a, *p* ≤ 0.05, *n* = 4). Second, the acute impact of the compounds on cell viability was measured and those that significantly reduced cell number were not considered further (Fig. 2b, *p* ≤ 0.05, *n* = 4). Third, we determined whether any of the compounds acted independently of the LDLR by measuring whether apoB was reduced in iPSC-derived hepatocytes generated from a hoFH patient^23^. The absence of LDLR activity renders the iPSC-derived hoFH hepatocytes unable to clear LDL by LDLR-dependent internalization^28^. Five out of the eleven tested compounds reduced apoB secretion without affecting cell viability in hoFH iPSC-hepatocytes (Fig. 2c, d). The reduction of apoB levels in the medium hoFH iPSC-hepatocytes indicates that the identified compounds act independently of the LDLR pathway.

**Fig. 3:**
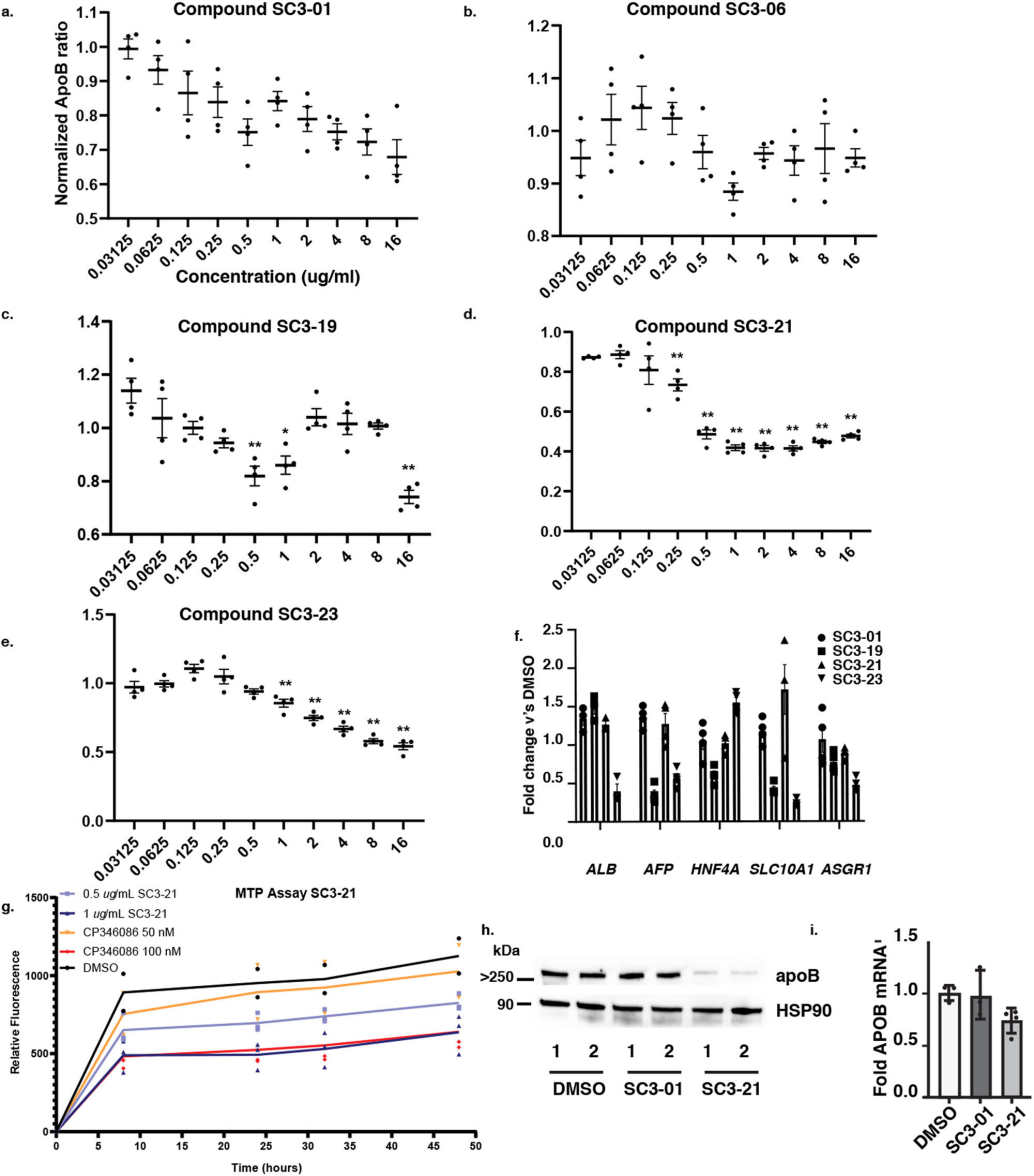
Characterization of compounds identified in primary screen. **a**–**e** Human iPSC-hepatocytes were treated with compounds SC3-01, -06, -19, -21, and -23 from 0.03125 to 16 µg/ml for 24 h. Culture medium from pre-drug and post-drug treatment were collected for human-specific apoB ELISA. Post-drug apoB: pre-drug apoB ratio from each treatment was determined and compared to DMSO-treated cells. All data are shown as mean ± SD of three replicates (*n* = 3). **f** Human iPSC-hepatocytes were treated with 2 µg/ml of compound SC3-01, -19, -21, and -23 for 24 h. Bar graph shows *ALB*, *AFP*, *HNF4α*, *SLC10A1*, and *ASGR1* mRNA levels that were determined by real-time qPCR. Fold change was calculated and normalized with vehicle only (*n* = 3). Data are shown as mean ± SD. All statistical analysis was performed using Student’s *t* test or one-way ANOVA followed by Dunnett’s test as appropriate; *p ≤ 0.05, **p ≤ 0.01. **g** Graph showing the impact on in vitro MTP activity of DL-21 (0.5 and 1.0 µg/ml) and the known MTP inhibitor CP346086 (50 and 100 nM) compared to vehicle (DMSO). **h** Immunoblots showing the steady-state level of apoB protein in human iPSC-hepatocytes treated with vehicle (DMSO) or 2 µg/ml SC3-01 or the MTP inhibitor SC3-21. HSP90 levels were used as a loading control. **i** Human iPSC-hepatocytes were treated with 2 µg/ml of SC3-01, SC3-21, or vehicle (DMSO) for 24 h. Bar graph shows normalized steady-state *APOB* mRNA levels determined by real-time qPCR (*n* = 4).

The five remaining candidates (SC3-01, SC3-06, SC3-19, SC3-21, and SC3-23) all displayed an ability to reduce apoB at 2 µg/ml. To differentiate between candidates, we performed 10-point dose response assays ranging from 0.03125 to 16 µg/ml of compound for 24 h in iPSC-hepatocytes and measured both apoB levels and cell viability (Fig. 3a–e and S1A–E). Compounds SC3-01, SC3-21, and SC3-23 all showed a dose-dependent reduction in apoB. Compounds SC3-06 and SC3-19 had an inconsistent impact with biphasic responses and so were excluded. Compound SC3-23 killed the cells at higher doses, while compounds SC3-01 and SC3-21 had no impact on cell viability (Fig. S1A, D). To exclude the possibility that a reduction in apoB reflected changes in the differentiation of iPSCs that could impact hepatocyte gene expression, we measured steady levels of mRNAs encoding *ALB*, *AFP*, *SLC10A1*, and *ASGR1* following treatment with 2 µg/ml of compounds SC3-01, SC3-19, SC3-21, and SC3-23 for 24 h (Fig. 3f). These markers were chosen because they are characteristically expressed in hepatocytes and together give an accurate and specific picture of the differentiation status of the cells^29^. Compounds SC3-19 and SC3-23 reduced the levels of a subset of these hepatocyte mRNAs, implying a broad impact on hepatocyte gene expression or differentiation and so were excluded. Compounds SC3-01 and SC3-21 had no impact.

**Fig. 4:**
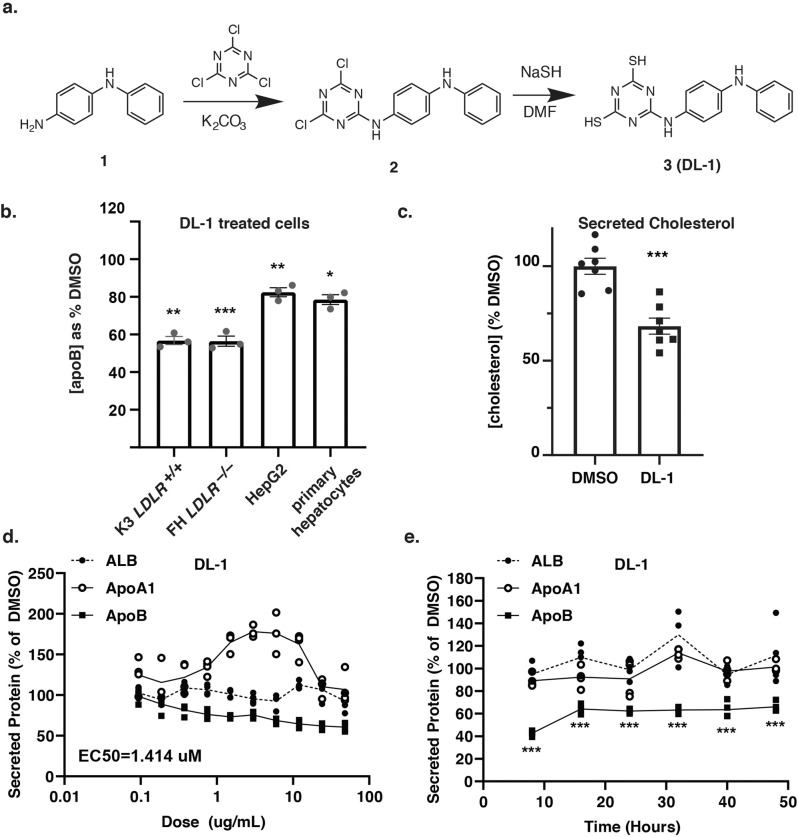
Compound DL-1 reduced apoB and cholesterol in hepatocyte culture medium. **a** The structure and synthesis of the lead compound DL-1 (formerly SC3-01). *N*-phenyl-1,4-benzenediamine (1) was reacted with cyanuric chloride in the presence of anhydrous potassium carbonate to form the intermediate compound (2). The latter reacted with sodium hydrosulfide (NaSH) in dimethylformamide (DMF) to form compound (3), DL-1. **b** Human iPSC-hepatocytes from a healthy donor (K3) and hoFH patient (JD4) along with human primary hepatocytes and HepG2 cells were treated with 2 µg/ml of DL-1 and compared to vehicle-treated cells (DMSO; 100%) for 24 h. ApoB levels in the medium were determined by ELISA (*n* = 3). **c** Graph showing the total cholesterol levels in the cell culture medium as determined by a cholesterol colorimetric assay. Pre:post treatment levels were compared to the DMSO-treated control group (*n* = 7). **d** Human iPSC-hepatocytes were treated with DL-1 with concentrations ranging from 0.0325 to 48 µg/ml for 24 h. Albumin, apoA1, and apoB levels in the culture medium were determined by ELISA assay (*n* = 3). **e** Cells were treated with 2 µg/ml of DL-1 from 8 to 48 h and pre-drug and post-drug medium was collected for analysis. The concentration of Albumin, apoA1, and apoB in the culture medium was determined by ELISA (*n* = 3). Secreted protein levels were compared to DMSO-treated cells. Data are shown as mean ± SD. All statistical analysis was performed using Student’s *t* test or one-way ANOVA followed by Dunnett’s test as appropriate; *p < 0.05, **p < 0.01.

The dramatic reduction in apoB after addition of compound SC3-21 was reminiscent of treating hepatocytes with drugs that blocked MTP. When we examined the structure of compound SC3-21, we found that it closely resembled other MTP inhibitors (Fig. S1F). We therefore compared the impact of compound SC3-21 on MTP enzymatic activity in vitro using a commercial assay. We found that compound SC3-21 was almost as potent an inhibitor as a previously characterized MTP antagonist CP-346086 (Fig. 3g). The identification of an MTP inhibitor validated the screening platform; however, given that other MTP inhibitors, such as Lomitapide, have undesirable side effects, we excluded compound SC3-21 from further study. We also tested SC3-01 in the MTP assay. Unfortunately, SC3-01 squelched the fluorescence-based assay even in the absence of MTP, preventing us from measuring its impact directly. Others have demonstrated that inhibition of MTP prevents the co-translational loading of lipid onto nascent apoB protein and as a consequence apoB is degraded by the proteasome^30–32^. We, therefore, performed immunoblot analyses to measure the impact of both SC3-01, and as a control the MTP inhibitor SC3-21, on steady-state intracellular apoB protein levels as an indirect assessment of any MTP inhibition (Fig. 3h). As expected, the MTP inhibitor SC3-21 markedly reduced intracellular levels of apoB. In contrast, no significant change in steady-state apoB levels were observed when cells were treated with SC3-01. We also examined the impact of SC3-01 or SC3-21 on *APOB* mRNA levels and found no significant change (Fig. 3i). Compound SC3-01 is 6-((4-(phenylamino)phenyl)amino)-1,3,5-triazine-2,4-dithiol (Fig. 4a) and its chemical structure is unrelated to any known MTP inhibitors. The absence of any structural similarity to known MTP inhibitors along with the observation that treatment does not impact steady-state apoB protein levels is consistent with the view that SC3-01 acts independently of MTP. From these data we conclude that SC3-01 reduces the amount of apoB protein detectable in the culture medium without affecting intracellular steady-state mRNA or protein levels.

### Compound DL-1 specifically reduced LDL-C secretion in hepatic cell lines

To avoid confusion with the nomenclature used in the parent library, the compound and derivatives were given the pre-fix DL (Duncan Lab). DL-1 was resynthesized from commercially available N-phenyl-1,4-benzenediamine (Fig. 4a; see Supplementary Methods for full synthesis scheme) and characterized by proton NMR spectroscopy and ESI TOF mass spectrometry with high accuracy. The synthesized DL-1 was found to be identical to the original compound in the SC^3^ library (Supplementary Methods). We confirmed that the resynthesized DL-1 had no impact on hepatocyte marker expression or cell viability (Fig. S2). To determine whether DL-1 could reduce secreted apoB levels across a range of hepatic cell models, we treated three additional cell types with the compound (Fig. 4b). We identified reductions of 40%, 44%, 23%, and 22% apoB in the medium of healthy donor (K3) iPSC-hepatocytes, hoFH patient iPSC-hepatocytes, HepG2 cells, and primary human hepatocytes, respectively.

VLDL/LDL is the major transporter of triglycerides and cholesterol from the liver and high levels are a risk factor for atherosclerosis^33^. We were unable to reliably measure triglyceride levels in iPSC-hepatocytes using a commercial assay. However, mean cholesterol levels were reduced by 32% in DL-1 treated cells compared to vehicle treatment (Fig. 4c, *p* ≤ 0.05, *n* = 7). This lowering of cholesterol therefore mirrored the observed reduction in apoB in DL-1 treated iPSC-hepatocytes.

We next investigated if DL-1 specifically reduced production of apoB or alternatively impacted protein secretion by hepatocytes more broadly. iPSC-hepatocytes were treated with DL-1 at a range of concentrations for 24 h, and the level of human apoB, apoA1, and albumin in the culture medium was evaluated by ELISA. DL-1 inhibited apoB secretion with an EC_50_ of 1.414 µM without significantly affecting the secretion of either Albumin or apoA1 into the culture medium (Fig. 4d). To determine the time-dependent response to DL-1, iPSC-hepatocytes were treated from 8 to 48 h. We observed a consistent reduction of apoB as early as 8 h after treatment. In contrast to apoB, neither apoA1 nor albumin secretion were affected by DL-1 throughout the entire 48-hour time course, confirming that the decrease in apoB was not due to a general reduction in protein secretion (Fig. 4e). It is noteworthy that apoB secretion was not completely blocked by DL-1 treatment, because examination of the kinetics of apoB levels in the culture medium revealed that it was consistently diminished to around 60% of control cells, but not eradicated even over time (Fig. 4e).

### Unlike MTP inhibitors DL-1 did not affect intracellular lipid accumulation

**Fig. 5:**
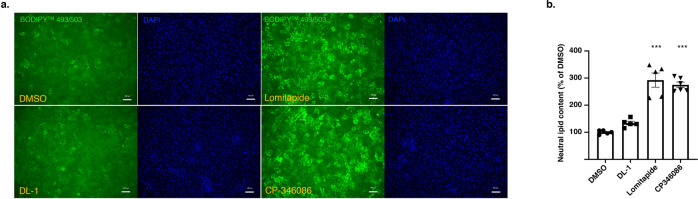
DL-1 did not increase hepatocyte lipid accumulation. Human iPSC-hepatocytes were treated with DL-1 (2 µg/mL), Lomitapide (20 nM), CP-346086 (20 nM), or vehicle (DMSO) for 72 h**. a** Hepatic neutral lipid was determined by staining with 2 µM of BODIPY 493/503 (green) for 15 mins and fixed with 4% PFA for microscopy. Nuclei were stained with DAPI (blue). Scale bar = 100 µm. **b** Bar graph representing the quantitative density measured by Keyence of neutral lipid staining. Fold change was determined by comparing to the DMSO-treated group. Data are shown as mean ± SD of five replicates (*n* = 5). Statistical analyses were performed using one-way ANOVA followed by Dunnett’s test; ***p < 0.001.

The current drugs that have been used for treatment of hoFH include Mipomersen, an *APOB* antisense oligonucleotide, and an MTP inhibitor called Lomitapide, both of which inhibit the assembly of VLDL within hepatocytes. Inhibiting VLDL particle formation can lead to the accumulation of intracellular hepatic triglyceride levels, leading to steatosis^34–36^. To determine if DL-1 treatment also caused hepatic lipid accumulation, we treated iPSC-hepatocytes for 72 h with either 2 µg/ml of DL-1 or MTP inhibitors CP-346086 and Lomitapide at 20 nM. Hepatic lipid accumulation was evaluated by staining for neutral lipid using BODIPY 493/503. As expected, cells treated with either CP-346086 or Lomitapide accumulated large numbers of lipid droplets which could be quantified by microscopy. In contrast to the MTP inhibitors, DL-1 treated iPSC-derived hepatocytes were indistinguishable from vehicle-treated control cells (Fig. 5a, b). These findings suggest that DL-1 inhibition of apoB does not drive lipid accumulation providing further support for the view that DL-1 acts independently of MTP. Moreover, these data imply that treatment could potentially avoid promoting the steatosis that is associated with Lomitapide and Mipomersen.

### Amino-triazine thiol core is critical for the activity of a 1,3,5-triazine-2-thiol family of DL compounds

The SC^3^ library contains 130,000 compounds representing over 10,000 diverse clusters with a threshold of 50% structural similarity according to the Tanimoto coefficient^37,38^. We computationally searched structures within the SC^3^ collection and retrieved an additional 20 compounds (DL-2 through DL-21) with ≥40% similarity to DL-1. To determine the activity of compounds and to begin to understand the structure-activity relationship, iPSC-hepatocytes were treated with each of the twenty analogs of DL-1 at 2 µg/ml for 24 h before measuring the impact on apoB levels in the medium. We observed that 15 compounds significantly reduced apoB, with a reduction ranging from 13% to 48% (Table 1).

**Table 1:** Activity of structurally related compounds within the SC^3^ collection.


Name	R1	R2	Similarity score (%)	Mean reduction (%)	S.D. (%)	*p*-value
DL-1	SH		100	34.1	±7.9	<0.001
DL-2	SH		91	15.0	±6.5	0.003
DL-3	SH		88	16.0	±6.3	<0.001
DL-4	SH		82	16.2	±6.3	<0.001
DL-5	SH		79	34.2	±12.8	<0.001
DL-6	SH		76	16.5	±8.6	0.002
DL-7			69	26.8	±8.1	<0.001
DL-8			64	35.1	±11.2	<0.001
DL-9	SH		64	31.1	±13.6	0.002
DL-10			62	48.6	±11.1	<0.001
DL-11	SH		56	7.5	±8.2	0.128
DL-12	SH		55	4.7	±12.4	0.496
DL-13			55	12.4	±13.9	0.069
DL-14	SH		52	6.2	±7.8	0.225
DL-15	SH		50	13.4	±6.0	0.003
DL-16	SH		50	23.1	±9.4	<0.001
DL-17	SH		50	23.6	±10.0	<0.001
DL-18	SH		50	33.1	±7.2	<0.001
DL-19	SH		48	33.5	±4.0	<0.001
DL-20	SH		45	-14.3	±9.0	0.004
DL-21	SH		43	13.5	±11.1	0.022

Since each of the compounds had distinct structures, we addressed whether specific chemical moieties correlated with activity. Such analysis revealed that the amino-triazine thiol core and the connection of a phenyl ring or long carbon chain are critical for compounds to reduce the apoB production. Small groups such as ethylamine, propan-2-amine, ethylethanamine, and morpholine were permissive but didn’t reduce apoB secretion levels. As shown in Table 1, the placement of R1 is not limited to a thiol group, because replacement with either ethylamine, ethylethanamine or propan-2-amine still conferred activity. Although it should be noted that the disulphur substituted compounds were the most represented in the SAR, they were not necessarily the most potent. Interestingly, replacing R1 with analine and R2 with methoxypropanamine (DL-14) significantly reduced activity, indicating that the positioning of at least one thiol group is critical. Furthermore, R2 was permissive to specific kinds of bulky or hydrophobic moieties, suggesting a non-charged binding surface for R2. To directly determine if the thiol group is essential for the apoB reduction, we tested chemical precursors of DL-1, DL-5, and DL-9 in which both thiols were replaced with chlorines (DL-1P, DL-5P, and DL9P, Supplementary Methods) and determined their efficacy for apoB reduction in iPSC-hepatocytes. Strikingly, no activity was observed in any of the chlorine substituted compounds (Table 2).

**Table 2:** Free thiol groups are critical for DL compound activity.

Name	Structure	Mean reduction (%)	S.D. (%)	*p*-value
DL-1		34.1	±7.9	<0.001
DL-1P		−3.8	±3.2	0.242
DL-5		34.2	±12.8	<0.001
DL-5P		6.3	±5.9	0.147
DL-9		31.1	±13.6	0.002
DL-9P		14.2	±13.3	0.117

**Fig. 6:**
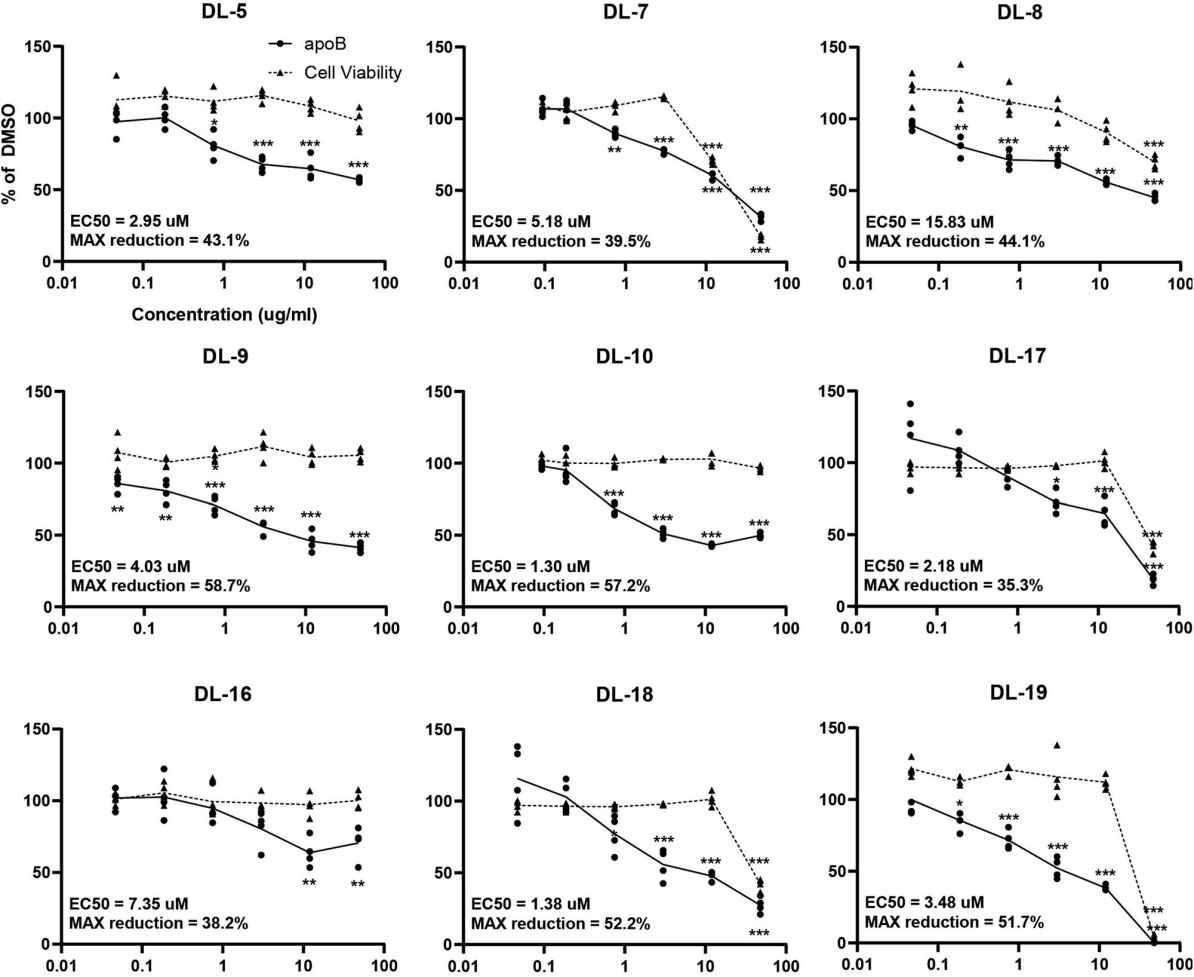
Dose-response analysis of DL-1 derivatives. Compounds that were structurally similar to DL-1 that had higher than a 20% apoB reduction in the cluster compound activity assay were picked for dose-response analyses. Dose response curves of apoB levels in pre- and post-drug medium were determined by ELISA and post-drug: pre-drug apoB ratios were calculated and normalized using the DMSO-treated group. EC_50_ for each compound was determined via Quest Graph™ EC_50_ Calculator. Cell viability was determined using the CellTiter-Glo luminescence assay. Data presented as mean ± SD of four replicates (*n* = 4). Statistical analysis was performed using one-way ANOVA followed by Dunnett’s test; *p < 0.05, **p < 0.01, ***p < 0.001.

Several key parameters, including compound potency, toxicity, drug-likeness, and medicinal chemistry friendliness, are generally evaluated to identify compounds with therapeutic potential. We first compared the potency of compounds to DL-1 by measuring the maximum apoB reduction and EC_50_. Nine compounds that decreased apoB in the culture medium by more than 20% were selected for dose-response studies. The results revealed that the EC_50_ ranges from 1.3 µM to 16 µM with the maximum reductions ranging from 58.7% to 39.5% (Fig. 6). Cell viability analysis showed that DL-7, DL-8, DL-17, DL-18, and DL-19 caused acute cell toxicity (Fig. 6), indicating that changing the functional groups can cause adverse effects at high doses. We further evaluated the drug-likeness and medicinal chemistry friendliness of the remaining five candidates (DL-1, DL-5, DL-9, DL10, DL-16), which had an EC_50_ within 10 µM without affecting acute cell viability. Using SwissADME, a model to predict small molecules’ pharmacochemistry^39^, DL-9 had a lower polar surface area, better solubility, higher predicated GI absorption, and predicted bioavailability compared to other active compounds although the predicted differences were modest (Supplementary Table 1). We also confirmed that like DL-1, DL-9 reduced apoB secretion into the medium without affecting steady-state protein or mRNA levels suggesting that the triazine thiols as a class perform similarly (Fig. S3).

### Toxicity and CYP450 functional tests in micropatterned co-cultured primary hepatocytes

**Fig. 7:**
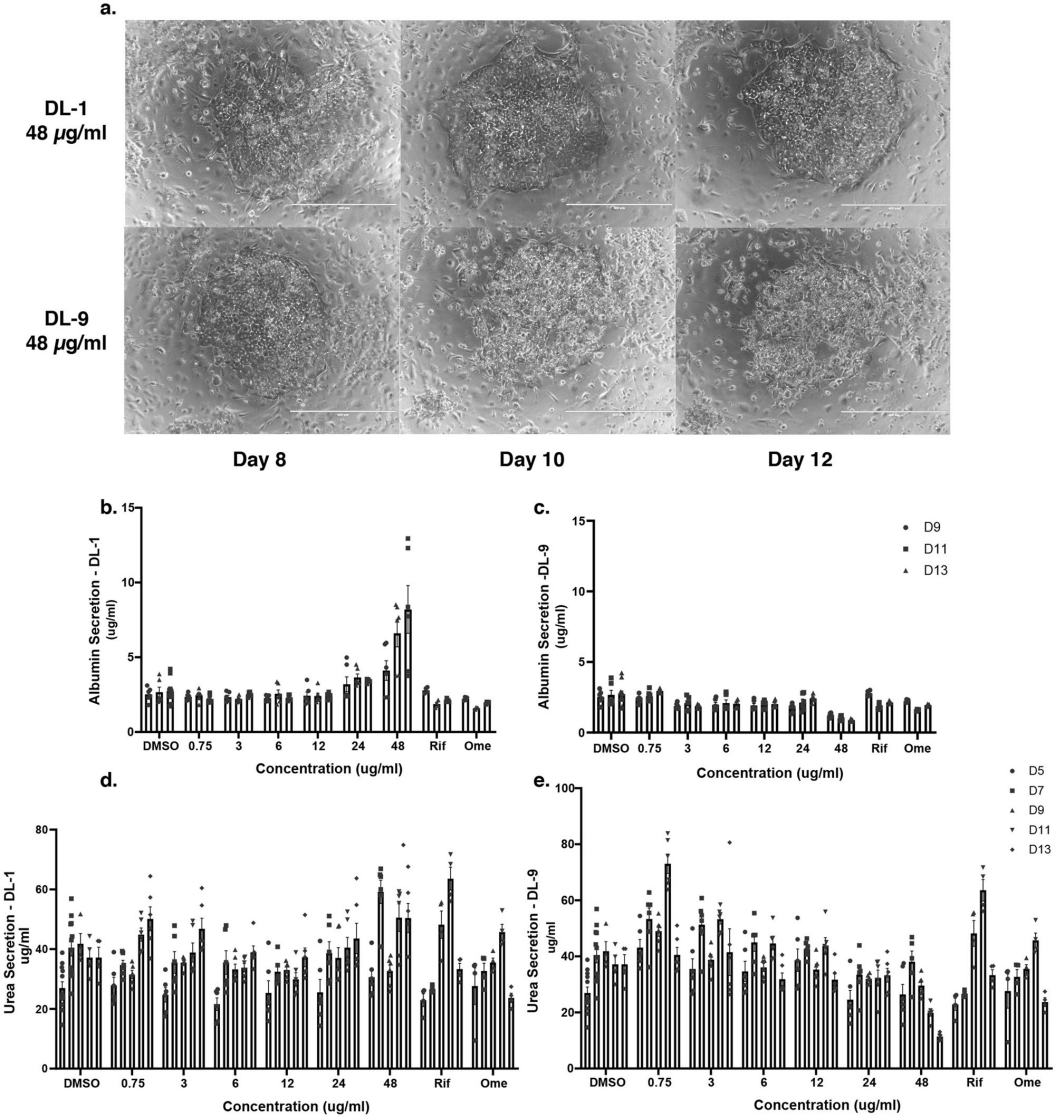
Toxicology of DL-1 and DL-9 in primary human hepatocytes. **a**–**e** Micropatterned co-cultures of primary human hepatocytes were treated DL-1 or DL-9 for up to 13 days at concentrations ranging from 0.75 to 48 µg/ml. Rifampin (25 µM) and omeprazole (50 µM) treated cells were included as controls. **a** Micrographs showing the impact of DL-1 and DL-9 on cell morphology after treatment for 8, 10, and 12 days with 48 µg/ml of each compound. **b**–**e** Bar graphs showing the level of Albumin (**b**, **c**) and Urea (**d**, **e**) in the medium of hepatocytes treated with 0.75, 3, 6, 12, 24, and 48 µg/ml of DL-1 or DL-9 for 9, 11, or 13 days (**b**, **c**) or 5, 7, 9, 11, and 13 days (**d**, **e**). Data presented as mean ± SD of three technical replicates (*n* = 3) and statistical analysis was performed using two-way ANOVA.

Although DL-1 and DL-9 showed no substantial impact on the acute viability of iPSC-hepatocytes, we have previously published that such cells have relatively low levels of a subset of cytochrome P450 enzymes including CYP3A4, which is critical for the metabolism of several drugs^40^. Micropatterned co-cultured primary human hepatocytes have been shown to maintain hepatocyte function for several weeks in culture and are one of the most robust culture models available to test hepatocyte response to small molecules^41^. Importantly, this platform has been used to successfully identify compounds that cause drug-induced liver injury^42^. We therefore used micropatterened co-cultured primary human hepatocytes to examine whether DL compounds affected hepatic function or were hepatotoxic. We examined both DL-1 and DL-9 because both compounds were predicted to have promising pharmacochemical properties. Six concentrations of each drug were tested (48 μg/ml, 24 μg/ml, 12 μg/ml, 6 μg/ml, 3 μg/ml, and 0.75 μg/ml) with treatments lasting 5, 7, 9, 11, and 13 days and micrographs collected on days 8, 10, and 12 (Fig. 7a). Changes in albumin and urea secretion were evaluated as markers of toxicity and hepatocyte function because they are more sensitive than intracellular ATP levels^42^. The activities of CYP3A4, 2A6, 2C9, and 1A2 were used as markers of metabolic induction or inhibition. Until day 8, both compounds were well tolerated by the primary hepatocytes at all concentrations tested (Figs. 7a and S4). However, as the treatment progressed beyond 8 days, DL-9 exhibited overt toxicity at the highest concentration tested (48 μg/mL), with cultures showing cell damage by 10 days of treatment (Figs. 7a and S4) and reduced albumin and urea secretion by day 11 (Fig. 7b–e). DL-9 also induced CYP3A4 and CYP2C9 in a concentration-dependent manner with activity peaking at around 12 μg/mL (Fig. S3). A modest induction of CYP2A6 was also observed. In contrast to DL-9, DL-1 did not induce hepatotoxicity or affect hepatic functions at any concentration, although we noted a minor inhibition of CYP3A4 at 48 μg/mL (Fig. S4).

### Triazine thiols are active in avatar mice with humanized livers

Although the cell model systems used to identify the triazine thiols are robust, confidence in the compounds would increase if they were efficacious in a physiological model. Both DL-1 and DL-9 are relatively insoluble compounds at 0.02 mgs/ml and 0.09 mgs/ml in PBS, respectively. The low solubility is problematic because it limits administration of the compounds in animals to routes that can accept large volumes. We therefore synthesized a compound called DL-27 that retained all active chemical groups associated with the triazine thiol class, but exhibited increased solubility at 1.8 mgs/ml (Fig. S5A). To ensure that DL-27 was active, we performed a dose response curve. The compound did not impact cell viability and reduced apoB by a maximum of 60% with an EC_50_ = 2.1 µM and so its efficacy in cells is comparable to DL-1 (Fig. S5B).

To identity a suitable animal model we performed a series of pilot experiments. *LDLR*^*–/–*^ mice (B6.129S7-Ldlr^tm1Her^/J) mice were given a triazine thiol at 10 or 20 mgs/kg/mouse for 14 days by intraperitoneal injection. While all mice (*n* = 9) survived compound treatment, surprisingly there was no impact of the triazine thiol on serum cholesterol or apoB levels. Although we had previously demonstrated that the triazine thiols were active in human cells, we considered the possibility that the mouse hepatocytes were unresponsive. To test this directly, we treated human iPSC-hepatocytes and primary mouse hepatocytes with vehicle, DL-1, DL-9, DL-27, or Lomitapide as a positive control (Fig. S5C). While all triazine thiols and Lomitapide reduced apoB in the human cells, only Lomitapide inhibited apoB production in the mouse hepatocytes. We conclude that mouse hepatocytes are resistant to triazine thiol treatment.

**Fig. 8:**
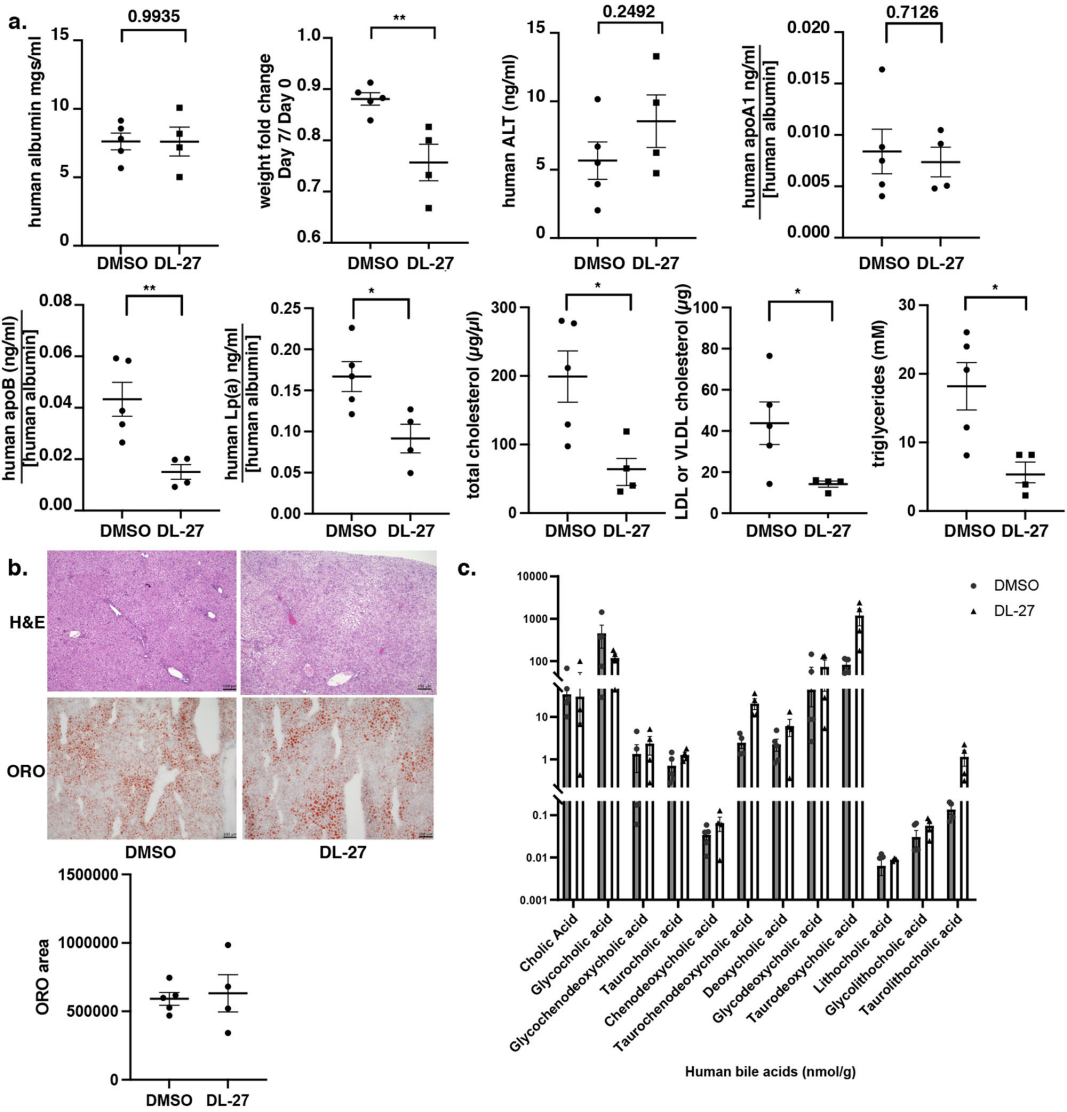
Triazine thiols are effective in liver-humanized mice. **a** Graphs showing weight changes during the course of the experiment and serum levels of albumin, ALT, apoA1, apoB, Lp(a), total cholesterol, LDL/VLDL, and triglycerides in liver-humanized mice following 7 days of treatment with either vehicle (DMSO; *n* = 5) or DL-27 at 20 mg/kg/day (*n* = 4). ELISA assays for albumin, apoA1, apoB, and Lp(a) were specific to human proteins and were normalized to human albumin levels to account for variation in the number of human hepatocytes present in each animal at the completion of dosing. Statistical analysis was performed using t tests; *p < 0.05, **p < 0.01, ***p< 0.001. **b** Micrographs showing H&E and Oil Red O stained sections through livers of liver-humanized mice treated with vehicle (DMSO) or the triazine thiol DL-27. Quantification of Oil Red O across representative liver sections in each mouse used in **a** was determined using ImageJ and shown graphically as the cumulative area of Oil Red O staining. **c** Bar graphs showing the quantity of 12 different bile acids in the livers of the humanized mice treated with DMSO or DL-27. Levels were determined using LC/MS metabolomics and displayed as a log scale.

The finding that the triazine thiols were only inhibitory to apoB production by human cells limited the use of mice as a model. However, Grompe and colleagues have previously demonstrated that mice with humanized livers are an effective model for the study of human lipoprotein metabolism^43^. We therefore partnered with Yecuris™ to address whether triazine thiols could lower apoB production in humanized livers. In all, 10 female liver-humanized *Fah*^-/-^*Rag2*^-/-^*Il2rg*^-/-^ NOD mice with ≥70% human hepatocyte repopulation were generated as described^44^. The hepatocyte donor was a 4–year old Hispanic female with a BMI = 16.2 (non-obese). All mice were weighed and grouped evenly based on albumin and/or body weight into 5 mice per cohort. Each mouse was dosed by intravenous retro-orbital injection (i.v.) with DL-27 in 5% DMSO at 20 mg/kg/mouse or 5% DMSO per day for 7 days. Terminal harvest took place 7 days post dosing when whole blood was collected and processed to serum. All mice enrolled into the study started losing weight on the second day of dosing and one animal in the treatment grouped died from unknown causes (Fig. 8). Human Albumin levels remained similar among animals confirming that the humanization of the livers remained stable at the end of the dosing (Fig. 8a). No significant difference in ALT levels was seen between groups suggesting that DL-27 was tolerated by the humanized livers. From the surviving mice, serum levels of apoB, Lp(a), apoA1, total cholesterol, LDL cholesterol, and triglycerides were measured at day 7. As predicted from the iPSC-hepatocyte data, serum levels of apoB, Lp(a), total cholesterol, LDL/VLDL cholesterol, and triglyceride were all significantly and substantially reduced in the DL-27 treated animals compared to the vehicle-treated group while apoA1, representing HDL, was unaffected. Livers from both the control and treatment groups were indistinguishable in H&E stained sections and no statistically significant difference in lipid accumulation was seen by Oil Red O staining (Fig. 8b).

Bile acids are synthesized by the liver from cholesterol^45^. As a consequence, bile acid synthesis has a prominent role in maintaining cholesterol homeostasis through cholesterol catabolism. We therefore reasoned that in the liver-humanized mice, inhibition of VLDL production by triazine thiol treatment may result in increases in bile acid synthesis^46^. A series of the 12 most prevalent human bile acids (Cholic acid, Glycocholic acid, Glycochenodeoxycholic acid, Taurocholic acid, Chenodeoxycholic acid, Taurochenodeoxycholic acid, Deoxycholic acid, Glycodeoxycholic acid, Taurodeoxycholic acid, Lithocholic acid, Glycolithocholic acid, and Taurolithocholic acid) were measured by LC/MS metabolomics (Creative Proteomics Metabolomics™; Fig. 8c). The majority of bile acids were unaffected, however, although we noted variation between individual animals, Taurocholic, Taurochenodeoxycholic, Taurodeoxycholic, and Taurolithocholic acids were all increased by as much as 10-fold in the compound-treated animals compared to controls.

## Discussion

In the current study, we used iPSC-derived hepatocytes to screen the SC^3^ small molecule library, which contains 130,000 proprietary compounds, with the goal of identifying molecules that could lower production of VLDL by the liver. By screening a structurally representative set of 10,000 small molecules we identified a compound called DL-1 that suppresses apoB production from hepatocytes with EC_50_ at low µM levels. DL-1 was found to be a member of a structurally related family of compounds containing a triazine-thiol group that were effective in both normal and hoFH patient-derived iPSC-hepatocytes confirming that the compounds act independently of the LDLR. We also found that triazine thiols were active in liver-humanized mice where they effectively lowered serum apoB, total cholesterol, triglycerides, VLDL/LDL-cholesterol, and Lp(a). Given that apoB and Lp(a) have been described as causal risk factors for atherosclerosis, it seems possible that the triazine thiols have therapeutic potential beyond the treatment of homozygous familial hypercholesterolemia^47^.

The core structure of the DL series is a 1,3,5-Triazine-2,4(1H,3H)-dithione that was originally used as a composite resin and surface treatment agent^48^. Our SAR analysis found that the thiol groups and a link between the triazine and aniline moieties are critical for the LDL-lowering effects. Compounds lacking the thiol groups showed no impact on apoB levels. Studies in a rat model revealed that both 6-ethoxycarbonyl-3-phenyl-1,3,5-triazabicyclo[3.1.0]hexane-2,4-dione and 2-ethoxycarbonyl-5-phenyl-1,3,5-triazine-4,6(1H,5H)-dione and 2-ethoxycarbonyl-5-(4-chlorophenyl)-1,3,5-trizine-4,6(1H,5H)-dione lowered circulating triglyceride and cholesterol levels by 40% after 16 days of treatment^49^. Although the mechanism of action was not defined, the authors proposed that the compounds acted on enzymes involved in de novo lipid synthesis^49^. This would be consistent with the observation that the compounds lack the critical thiol groups and implies a mode of action that is distinct from the DL compounds.

Hepatic VLDL assembly and secretion is regulated at multiple checkpoints offering options to inhibit the production of VLDL by the liver. Mipomersen, an antisense oligonucleotide that specifically binds to *APOB* messenger RNA to block translation of the gene, showed a decrease in circulating levels of apoB-containing atherogenic lipoproteins^50^. Lomitapide, a small molecule that inhibits MTP activity, prohibits VLDL particle lipidation and induces ER-associated degradation of apoB thereby inhibiting VLDL secretion^30^. However, mutations in the *apoB* gene can cause familial hypobetalipoproteinemia and mutations in *MTTP*, which encodes MTP, cause abetalipoproteinemia. In both cases steatosis is commonly observed in patient’s livers^34,51,52^. Similarly, in mouse models, lack of apoB protein or MTP activity causes abnormal hepatic lipid accumulation^53–56^. In line with the genetics, both Mipomersen and Lomitapide treatment can cause hepatic fat accumulation and so must be used cautiously^57,58^. The accumulation of lipid caused by MTP loss of function could be recapitulated using hepatocytes derived from abetalipoproteinemia iPSCs^52^. Using the iPSC-hepatocyte model, we found that MTP inhibitors also caused an increase in intracellular lipid content. In contrast, treating the cells DL compounds had no impact on lipid levels. These data suggest that the pathway through which DL compounds regulate apoB secretion is independent of ERAD, which is consistent with our finding that treatment of iPSC-hepatocytes with DL compounds had no impact on steady-state apoB protein or mRNA levels.

Drugs that target VLDL production or transport after ER-related post-translational regulation could potentially circumvent the steatosis associated with the degradation of apoB by ERAD. Post-ER compartments have drawn increasing interest for their role in presecretory VLDL regulation in recent years^59–61^. Post-ER, presecretory proteolysis occurs after VLDL is formed; in contrast to ERAD, PERPP does not disrupt the lipidation of apoB^61,62^. Pan and colleagues revealed that docosahexaenoic acid stimulated oxidative modification and aggregation of apoB after leaving the ER. Before the aggregated apoB exits the cell, it is transported to the autophagosome and is slowly degraded as a late-stage quality control process^63^. In another study, hepatic sortilin (SORT1) was identified as a critical lysosomal sorting protein that bound intracellular apoB-containing particles within the Golgi and directed them to the lysosome for degradation^64^. The study also revealed that autophagy is required for sortilin-induced lysosomal apoB degradation^65–67^. More recently, it has been shown that during normal conditions transport of apoB to the lysosome is modest^68^. However, under high fat or ER stress conditions sortilin enhances lysosomal trafficking of apoB to promote VLDL degradation. While speculative, it seems possible that the triazine thiols could promote lysosomal targeting and experiments are currently underway to address this potential mechanism.

In summary, the present study provides the pharmaceutical composition of compounds, comprising an 1,3,5-Triazine-2,4(1H,3H)-dithione, that can reduce hepatic LDL-C secretion by human hepatocytes. We propose that the DL compounds could be used as an alternative treatment for hoFH patients and individuals who are resistant or intolerant to statins. Although the mechanism of action appears to be downstream of ERAD checkpoints, further work is required to identify the target with which the compounds interact. Once identified, the target will likely reveal pathways for future pharmaceutical development and optimization. Moreover, we have validated the feasibility of utilizing the iPSC-hepatocytes platform to perform large-scale phenotypic screens. Combined with advanced genome-editing techniques and the availability of iPSCs from patients, we believe that the iPSC-hepatocyte screening platform is a cost-effective drug discovery tool to identity therapies for rare liver diseases.

## Methods

### Induced pluripotent stem cell culture and hepatocyte differentiation

Use of human iPSCs was approved by the MUSC Stem Cell Research Oversight Committee. Human K3 iPSCs were generated previously and have been described elsewhere^40^. The hepatocyte differentiation protocol has been published elsewhere^21,25,40^ with minor modifications. Briefly, human iPSCs were seeded on Matrigel-coated tissue culture plates with a density of 7 × 10^5^ cells/ml to form a monolayer. Cells were induced to form definitive endoderm in RPMI 1640 Medium (Invitrogen, Waltham, MA) supplemented with 2% B-27 Supplement minus insulin (Invitrogen), 10 ng/mL of BMP4 (Invitrogen), 20 ng/mL FGF2 (Invitrogen), and 100 ng/mL Activin A (Invitrogen) for two days, followed by 100 ng/mL Activin A for three days. Definitive endoderm was then converted to hepatocyte progenitor cells by addition of BMP4 (20 ng/mL) and FGF2 (10 ng/mL) for an additional 5 days. Immature hepatocytes were generated by the inclusion of Hepatocyte Growth Factor (20 ng/mL) (Invitrogen) for 5 days. Cells were finally induced to mature to hepatocyte-like cells by the addition of Oncostatin M (20 ng/mL) (Invitrogen) for 5 days.

### Drug screen, compound library, and apoB assays

A detailed step-by-step description of the screening procedure has been published previously^25^. K3 iPSCs were differentiated to hepatocyte-like cells in 96-well plates. 24 h after a medium change, a pre-drug treatment sample was collected before the addition of drugs in fresh medium. A 10,000-compound screening set with scaffolds representative of the entire SC^3^ library was then screened to identify novel inhibitor scaffolds. The average physicochemical properties of the SC^3^ are MW ~400 g/mol, cLogP~2, and ~7 rotatable bonds. In terms of diversity, the SC^3^ has more than 10,000 clusters with a threshold of 50% similarity and ~70,000 clusters at 80% similarity. The SC^3^ 10,000 compound screening set therefore represents 10,000 clusters of compounds within the SC^3^ library. Compounds from the screening set were added for 24-h at 2 µg/mL, with DMSO at a final concentration of 0.2% and post-drug culture medium was then harvested for analysis. ApoB levels in the collected medium were determined by ELISA in both pre-and post-drug treated samples. We have previously demonstrated the screening assay had a Z′ = 0.73 and so was compatible with high throughput screening^25^. The apoB levels in the pre-drug and post-drug medium were combined and expressed as a delta-apoB ratio (post-drug [apoB]: pre-drug [apoB]).

### Synthesis of compounds

A detailed description of the synthesis of DL-1, DL-5, and DL-9 is included the Supplementary Methods section.

### Enzyme-linked immunosorbent assays

Human apoB levels were determined using a commercial sandwich ELISA (3715-1H-6; Mabtech, Cincinnati, OH), and the signal was detected using the HRP-TMB system. The concentration of apoB was calculated using a standard curve with a four-parameter logistic regression model. Human apoA1 levels were determined by ELISA (3710-1H-6; Mabtech) following the manufacturer’s instructions. Human Albumin levels in cell culture supernatants were measured by commercial ELISA (E88-129; Bethyl Laboratories, Montgomery, TX) following the manufacturer’s instructions. Lp(a) levels were measured using the Human Lipoprotein A SimpleStep ELISA kit (ab212165; Abcam, Cambridge, UK) as described by the manufacturer. Mouse serum apoB levels were measured using the Mouse ApoB Simplestep ELISA Kit as described by the manufacturer (ab230932; Abcam, Cambridge, UK). Total cholesterol and LDL/VDL levels were measured in humanized mice using the Cholesterol Assay Kit - HDL and LDL/VLDL (ab65390; Abcam, Cambridge, UK) and triglyceride levels measured using Triglyceride Assay Kit, Quantification (ab65336; Abcam, Cambridge, UK).

### Cell viability assay

Acute cell viability was performed using the CellTiter-Glo® luminescent cell viability assay following the manufacturer’s instructions (Promega, WI, US). Experimental and control groups were processed identically. Luminescence was determined using a SYNERGY/HTX multi-mode plate reader (BioTeck, Winooski, VT).

### Microsomal transfer protein activity assay

MTP activity was determined using commercially available MTP activity assay kit (#MAK110, Sigma). 5 µg of purified rat MTP (#RB-RMTP, Roar biomedical, New York, NY) was incubated with MTP assay agent with or without 0, 3, 12, and 48 µg/mL of DL-1 and DL-9 at 37 °C for 1 hour. Fluorescence value (λex = 465/λem = 535 nm) was measured and MTP activity was determined by normalizing the fluorescence value with MTP assay agent only as the sample blank.

### Cholesterol quantification

Cell Culture medium was harvested from human iPSC-hepatocytes before and after 24 h of compound (2 µg/mL) or vehicle treatment. Pre-drug and post-drug media were concentrated using Amico® Ultra 10 K Centrifugal Filters (#Z740171, Sigma-Aldrich, St. Louis, MO), and total cholesterol levels were determined using the colorimetric cholesterol assay kit (#MAK043, Sigma-Aldrich) following the manufactural instructions. Post:Pre-drug ratios were determined and normalized to vehicle treatment group.

### Metabolic labeling, immunoprecipitation, and immunoblot

Human iPSC-hepatocytes were pretreated with 3, 12, and 48 µg/ml of compound for 2 h. Cells were then starved with methionine-free DMEM medium for 30 mins before pulse-labeling with [35 S]-Met DMEM medium for 10 mins. The medium was replaced with growth medium containing 0, 3, 12, and 48 µg/ml of compound for 2 h, and cell lysates were collected. Immunoprecipitation of cell lysates was performed using protein G magnetic beads (ThermoFisher, Waltham, MA) and anti-apoB (LDL20/17, Mabtech) antibodies. Eluted protein samples were separated using 4-15% acrylamide gradient SDS-PAGE. The radio-labeled signal was developed using FLA 9000 Typhoon Storage PhosphorImager (GE Healthcare, Chicago, IL). Expression levels were quantified via integrated intensity normalized by 5% of input of each sample. For Western blot analysis, iPSC-hepatocytes were pretreated with or without 100 nM bafilomycin A1 (Tocris, Minneapolis, MN) for 2 h followed by 3 µg/ml of compound treatment. Cells were harvested in RIPA buffer and protein concentrations were determined by BCA assay (ThermoFisher). Protein (20 µg) was separated by SDS-PAGE followed by transfer to PVDF membranes. Membranes were probed with anti-apoB (LDL 20/17, Mabtech), and GAPDH (ab128915, Abcam, Cambridge, UK). Signals were detected using HRP-conjugated secondary antibodies (Abcam). Band density of apoB protein was quantified and normalized to GAPDH using ImageJ Lab software.

### Immunostaining

For hepatocyte marker expression analysis, human iPSC-hepatocytes were treated with 2 µg/ml of compound for 24 h. Cells were fixed in 4% paraformaldehyde and permeabilized with 0.1% Triton-X-100. Cells were incubated with primary antibodies (ALB, 1:1000, Dako, Santa Clara, CA; HNF4-a, 1:1000, Santa Cruz, Dallas, TX) overnight at 4˚C. Samples were washed and then incubated with secondary antibodies (Alexa Flour 488 goat anti-rabbit, 1:1000, Invitrogen; Alexa Flour 594 rabbit anti-mouse, 1:2000, Invitrogen). Images were taken using Bio-Rad Zoe^TM^ Fluorescent Cell Imager (Bio-Rad, Hercules, CA). For intracellular apoB localization, iPSC-hepatocytes were treated with 0, 3, 12, 48 µg/ml of compound for 24 h. Immunostaining was completed as described above with primary antibodies (anti-apoB 1:100, Mabtech; anti-LAMP1, 1:100, Cell Signaling, Danvers, MA, anti-SORTILIN, 1:100, Abcam). Images were taken using KEYENCE BZ-800 and Leica SP8 confocal microscopy. Integrated fluorescent intensity was normalized by total cell counts, as determined by DAPI staining.

### Quantitative real-time PCR analysis

Human iPSC-hepatocytes were treated with 2 µg/ml of compound for 24 h. RNA was isolated from compound-treated and control iPSC–HLCs using the RNeasy Mini Kit (#74106, Qiagen, Hilden, Germany). Genomic DNA was removed using the TURBO DNA-free™ Kit (#AM1907, ThermoFisher,). First-strand cDNA was synthesized using MMLV Reverse Transcriptase (#28025-013, ThermoFisher,). Quantitative real-time PCR was performed on a BioRad CFX384 real-time PCR machine using Taqman polymerase. Primetime primer sequences (IDT Coralville, IA) are listed in Supplementary Table 2.

### Toxicity and Cytochrome P450 (CYP) functional tests in micropatterned co-cultured primary hepatocytes

Cryopreserved primary human hepatocytes were obtained from Triangle research labs, (Durham, NC): HUM4145 (1 year and 8 months old male Caucasian). Micropatterned co-cultures were created as previously described^41^.

Cell morphology was monitored using an EVOS^®^FL cell imaging system (Life Technologies, Carlsbad, CA) with standard 10× phase contrast objective. Culture supernatants were assayed for albumin using a competitive enzyme-linked immunosorbent assay^69^ or a sandwich ELISA (Bethyl Laboratories). Urea concentration in supernatants was assayed using a colorimetric end-point assay utilizing diacetyl monoxime with acid and heat (Stanbio Labs, Boerne, TX). Absorbance values were quantified on the Synergy H1 multi-mode plate reader (BioTek).

CYP3A4 and CYP2C9 enzyme activities were measured by incubating the cultures with luciferin-IPA (3 µM) or luciferin-H (100 µM) substrates (Promega Life Sciences, Madison, WI) for 1 hour and 3 h, respectively. The metabolite, luciferin, was quantified via luminescence detection on the Synergy H1 multi-mode plate reader. CYP1A2 and CYP2A6 activities were measured by incubating the cultures with 5 µM 7-ethoxyresorufin or 50 µM coumarin (Sigma-Aldrich) for 3 h and 1 hour, respectively. The metabolites, resorufin and 7-hydroxycoumarin (7-HC), generated from 7-ethoxyresorufin and coumarin, respectively, were quantified via fluorescence detection (excitation/emission: 550/585 nm for resorufin, and 355/460 nm for 7-HC) on the Synergy H1 reader^70^.

For drug-mediated CYP induction studies, MPCCs (7 days old) were exposed to serum-free culture medium containing rifampin (25 µM), omeprazole (50 µM), or dimethylsulfoxide (DMSO) alone (0.1% v/v) (Corning). CYP3A4, CYP2C9, CYP2A6, and CYP1A2 enzyme activities were quantified after 2 days and 4 days of drug exposure using assays as described above.

For drug toxicity studies, MPCCs (7 days old) were treated in serum-free medium to six concentrations of each drug (48 μg/mL, 24 μg/mL, 12 μg/mL, 6 μg/mL, 3 μg/mL, and 0.75 μg/mL) every other day for a total of 6 days. This timing was previously shown to increase sensitivity for drug toxicity detection without increasing the false positive rate^42^. All compounds were dissolved in 100% DMSO and the final DMSO concentration in the culture medium was kept at 0.2% (v/v).

### Humanized mice

Description of methods provided by Yecuris™. Yecuris Corporation (Oregon) provided 10 adult (older than 6 weeks) female liver-humanized *Fah*^-/-^*Rag2*^-/-^*Il2rg*^-/-^ NOD mice on a C57bl/6 background with ≥70% human hepatocyte repopulation as described^44^. The animals were housed in an IACUC accredited facility at the Yecuris™ facility in Oregon. General procedures for animal care and housing were as described in the Guide for the Care and Use of Laboratory Animals, National Research Council, Yecuris™ IACUC Policy and Yecuris™ General Mouse Handling Care and Euthanasia. Cages were changed every two weeks and the testing facility was sanitized weekly. All mice enrolled in the study were removed from 2-(2-Nitro-4-trifluromethylbenzoyl)-1,3-cyclohexanedione (Nitisinone, NTBC) for ≥20 days and SMX/TMP for ≥3 days prior to dosing. The hepatocyte donor (Donor: HHF04030) was a 4-year-old Hispanic female with a BMI = 16.2 (non-obese). Liver-humanization was evaluated ≤ 7 days prior to start of study by human albumin ELISA. All mice were weighed and grouped evenly based on albumin and/or body weight into 5 mice per cohort. Each mouse was dosed with DL-27 in 5% DMSO at 20 mg/kg/mouse or 5% DMSO once a day, every day for 7 days and ending 1 day before harvest. For dosing, the mice were anesthetized with vaporized isoflurane and dosed retro-orbitally. On day 0 and terminal harvest, whole blood was collected retro-orbitally and processed to serum. Terminal harvest took place 7 days post dosing. All animals were weighed and anesthetized using a ketamine cocktail (12.5 µL/g BW). All mice enrolled into the study started losing weight on the second day of dosing and one animal in the treatment grouped died from unknown causes.

### Bile acid measurement

Analyses of bile acids was performed by Creative Proteomics Metabolomics (Shirley, NY) on flash frozen liver of liver-humanized mice. Method was provided by Creative Proteomics Metabolomics. An Agilent 1290 UHPLC system coupled to a Sciex 4000 Qtrap mass spectrometer was used. The MS instrument was operated in the multiple reaction monitoring mode with negative-ion detection. A Waters BEH C18 column (2.1 × 150 mm, 1.7 μm) was used and the mobile phase was (A) 0.01% formic acid in water and (B) 0.01% formic acid in acetonitrile for binary-solvent gradient elution. A mixture of standard substances containing all the targeted BAs was dissolved in 80% methanol to have a concentration of 20 μΜ for each bile acid. This solution was further diluted step by step at a same dilution ratio of 1 to 4 (v/v) with the same solvent to have 10-point calibration solutions. In all, 100 μL of each solution was then mixed with an equal volume of an internal standard (IS) solution containing 14 D-labeled bile acids. In all, 10 μL of each resultant solution was injected to run UPLC-ESI-MS/MS with the scheduled MRM. Linear regression calibration curves were constructed with the analyte-to-internal standard peak area ratios (As/Ai) versus molar concentrations (nmol/mL).

### Statistics and reproducibility

For the primary screen, a *z*-score was generated for each compound using the delta-apoB ratio with the standard deviation of the delta-apoB ratios. Primary hits were validated in secondary replicate experiments (biological replicates *n* = 4), and significance was determined by a Student’s *t* test. Other data were reported as mean ± SD unless otherwise noted, with the number of each independent experiment provided in the figure legends. Each experiment was performed in at least three replicates and the statistical significance was calculated by two-tailed Student’s *t* tests or one-way ANOVA followed by Dunnett’s test for multiple comparison. A *p*-value <0.05 was considered significant. Raw data and statistics used in the manuscript are available in the Supplementary Data section.

### Reporting summary

Further information on research design is available in the Nature Portfolio Reporting Summary linked to this article.

## Supplementary information

Supplemental Information

Description of Additional Supplementary Files

Supplementary Data

Reporting Summary

## Data Availability

The raw datasets generated or analyzed during the current study are available for download as Supplementary data.
