# Supplementary material for: A human iPSC-derived hepatocyte screen identifies compounds that inhibit production of Apolipoprotein B
Source: Commun Biol. 2023 Apr 24;6:452. doi: 10.1038/s42003-023-04739-9 (PMC10125972; doi:10.1038/s42003-023-04739-9)
Supplement: Supplementary file 1 — Supplemental Information [file 42003_2023_4739_MOESM1_ESM.pdf]

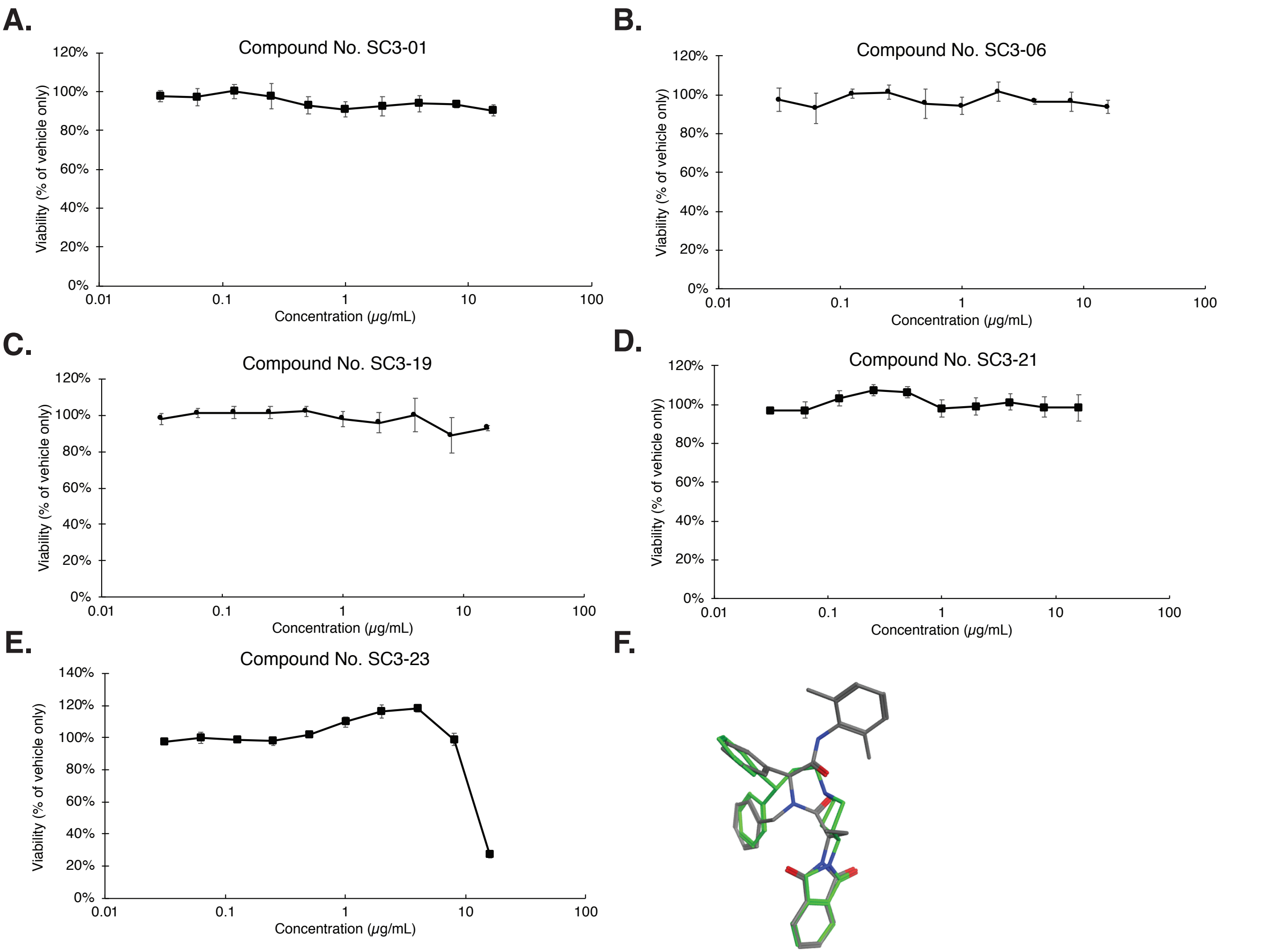

Figure S1. Viability of ipSC-hepatocytes treated with compounds from primary screen. (A-E) Graphs showing the viability of cells treated with 0.03125 to 16 µg/ml for 24 hours of compounds 1, 6, 19, 21, and 23 compared to DMSO control. All data are shown as mean ± SD of three replicates (n=3). (F) Overlay of the chemical structures of compound 21 (grey) with MTP inhibitor BMS200150 (green).

Supplemental Figure 2.

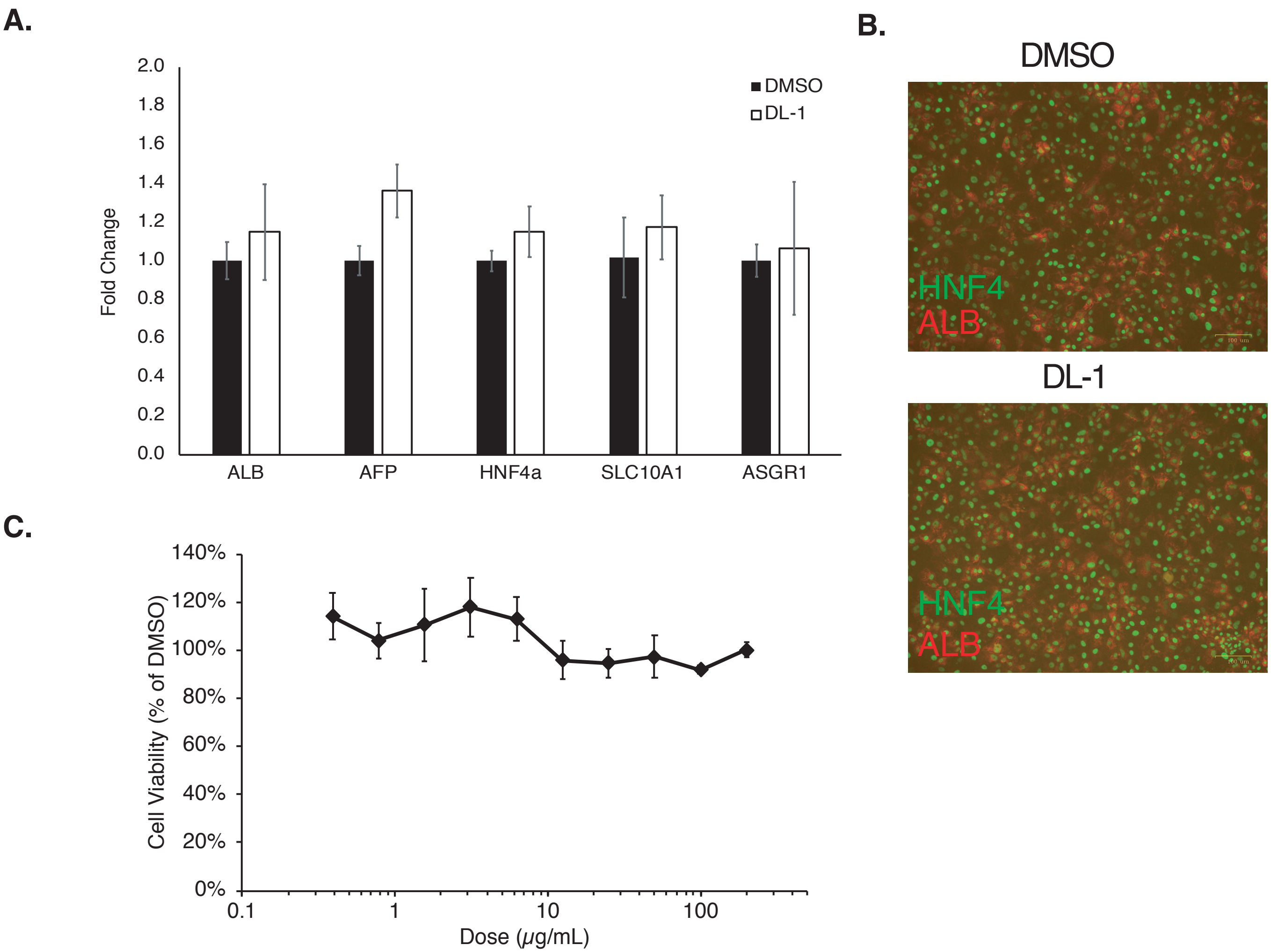

Figure S2. DL-1 did not affect hepatocyte gene expression or cell viability. (A, B) Human iPSC-hepatocytes were treated with 2 μg/ml of DL-1 for 24 hours. (A) Bar graph showed mRNA level of hepatic markers (ALB, AFP, HNF4α, SLC10A1, ASGR1), as determined by real-time qPCR. Fold change was calculated and normalized with vehicle only (n=3). (B) Immunofluorescence staining to determine HNF4A and ALB protein expression in response to DL-1 treatment.
Scale bar = 100 μm. (C) Human iPSC-hepatocytes were treated with DL-1 ranging from 0.4 μg/ml to 200 μg/mL, for 24 hours. Bar graph shows the average ATP content for each condition as a measurement of cell viability determined by using an ATP-based luminescent cell viability assay CellTiter Glo. Data are shown as mean ± SD (n=4).
Statistical analysis was performed using one-way ANOVA followed by Dunnett's test.

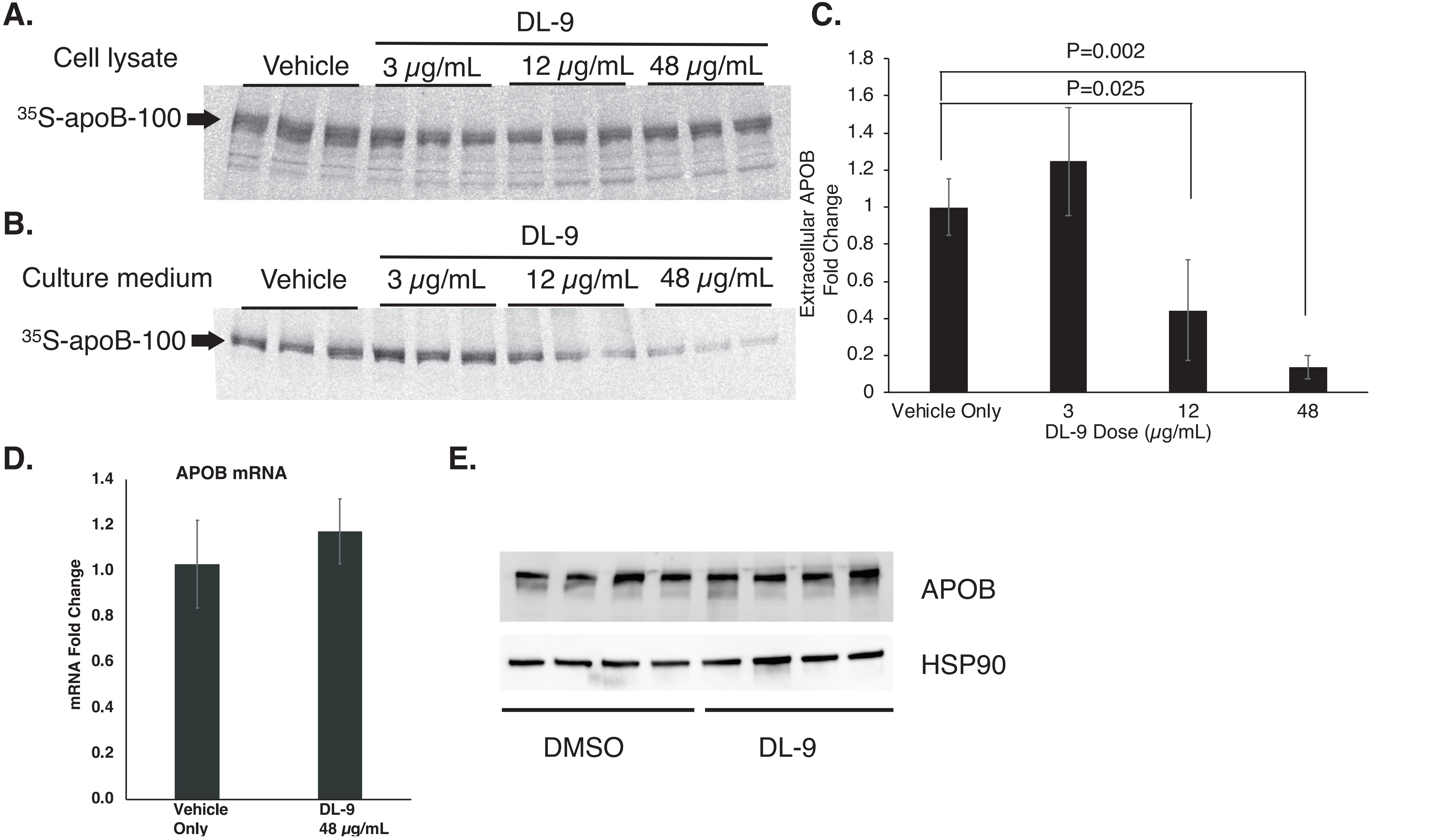

Figure S3. DL-9 reduces apoB production without impacting steady protein or mRNA levels. (A - C) IPSC-hepatocytes were pre-treated with 3, 12, and 48 µg/mL of DL-9 for 2 hours, and cells were pulse-labeled with [35S]-methionine DMEM for 10 minutes. Medium was replaced with HCM again containing 3, 12, and 48 µg/mL of DL-9 for 120 minutes. 35S-apoB in the cell lysate (A) and culture medium (B) was determined by immunoprecipitation using anti-apoB antibody. (C) Bar graph showing density of bands in culture medium that were quantified by phosphorimager analyses and normalized using 5% of input, (n=3). (D) Human iPSC-hepatocytes were treated with 48 µg/mL of DL-9 or vehicle (DMSO) for 24 hours. Bar graph showed apoB steady-state mRNA levels determined by real-time qPCR. Fold change was calculated and normalized with vehicle only (n=5). (E) Immunoblots showing the steady-state level of apoB protein in human iPSC-hepatocytes treated for 24 hrs with vehicle (DMSO) or 2 µg/ml DL-9 21. HSP90 levels were used as a loading control. All bar graphs are shown as mean ± SD. All statistical analysis was performed using Students t-test or one-way ANOVA followed by Dunnett's test as appropriate.

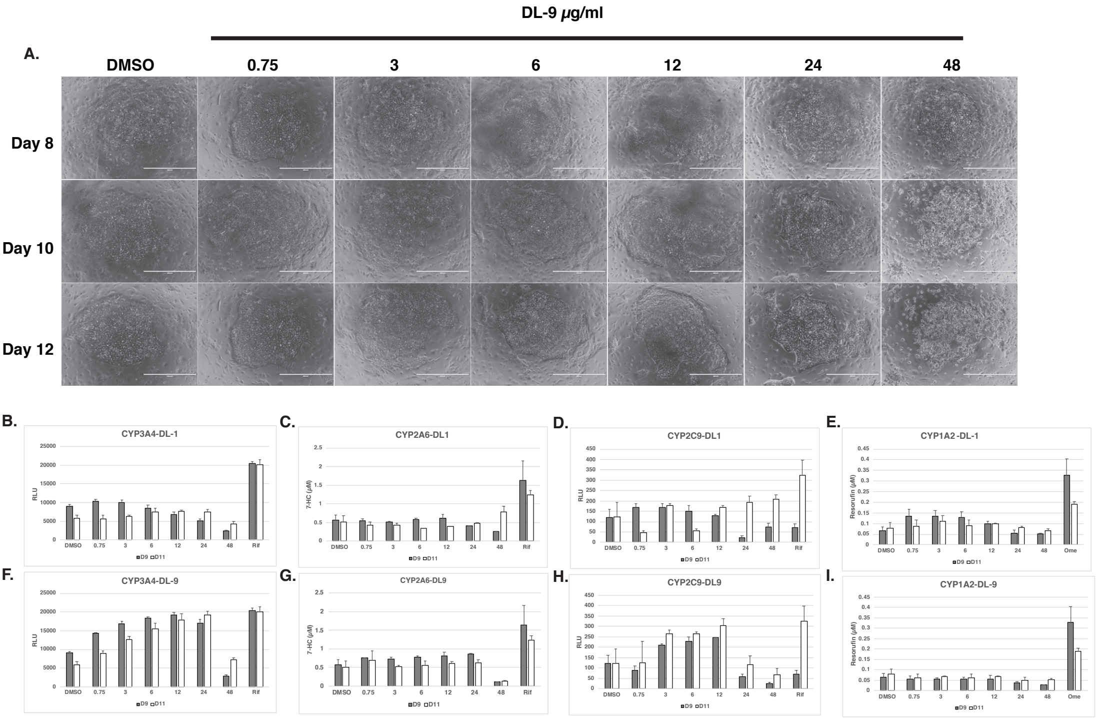

Figure S4. DL-9 induced hepatocyte damage and toxicology. (A) Micropatterned co-cultures of primary human hepatocytes were treated DMSO or DL-9 for 8, 10 and 12 days at 0.75, 3, 6, 12, 24 and 48 μg/ml. Micrographs show the impact of DL-9 on cell morphology and viability. (B - I) Bar graphs showing the level of CYP3A4 (B, F), CYP2A6 (C, G), CYP2C9, (D, H) and CYP1A2 (E, I) in the medium of hepatocytes treated with 0.75, 3, 6, 12, 24, and 48 μg/ml of DL-1 (B - E) or DL-9 (F - I) for 9 (grey bar) or 11 (white bar) days. Rifampin (25 μM) and Omeprazole (50 μM) treated cells were included as controls where appropriate. Data presented as mean ± SD of three technical replicates (n=3) and statistical analysis was performed using two-way ANOVA.

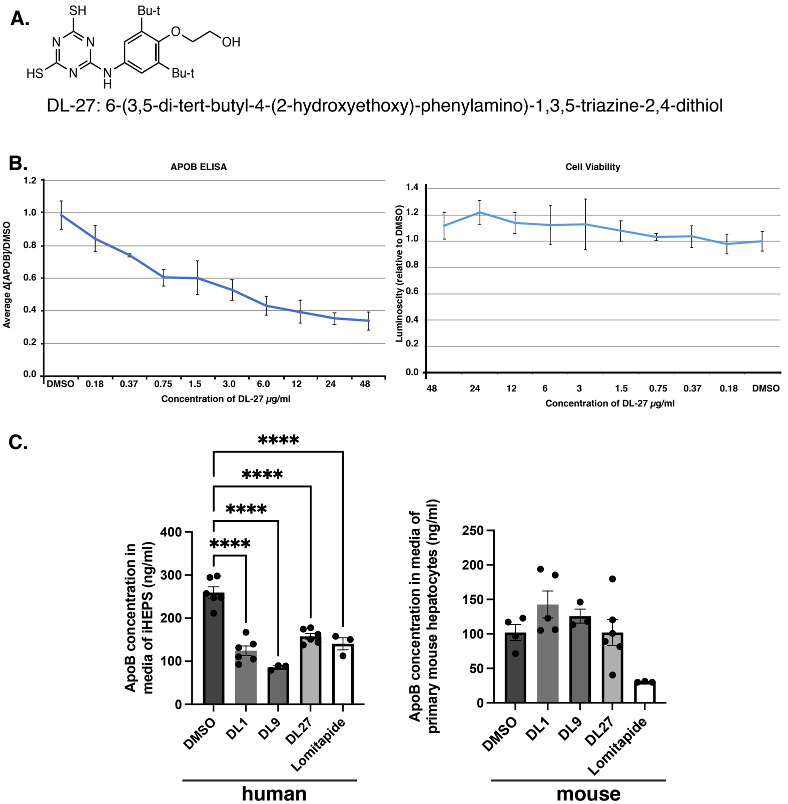

Figure S5. Mouse primary hepatocytes are resistant to triazine thiols DL-1, DL-9 and DL-27. (A) Chemical structure of DL-27 which has improved solubility. (B) Dose response curve for DL-27 was determined by ELISA and post-drug: pre-drug apoB ratios were calculated and normalized using the DMSO-treated group. The DL-27 EC50 was determined after converting to molarity. Cell viability was determined using the CellTiter-Glo luminescence assay. Data presented as mean ± SD of four replicates (n=4). (C) Bar graphs showing response of iPSC-hepatocytes (left panel) or primary mouse hepatocytes (right panel) following treatment for 24 hrs with DMSO, DL-1, DL-9, DL-27 or Lomitapide as a positive control. Statistical analysis was performed using one-way ANOVA; * p<0.05, ** p<0.01, *** P<0.001. **** P<0.0001.

Supplemental Table 1. Predicted medicinal chemistry of DL-1 and derivatives.

Molecule	MW	#Rotatable bonds	TPSA	Consensus Log P	Silicos-IT class	GI absorption	log Kp (cm/s)	Bioavailability Score
DL-1	327.4	4	140.3	3.2	Poorly soluble	Low	-5.2	0.55
DL-5	305.2	2	128.3	3.14	Moderately soluble	High	-5.27	0.55
DL-9	362.5	4	70.93	3.96	Moderately soluble	High	-4.39	0.85
DL-10	444.6	8	203.1	3.13	Poorly soluble	Low	-5.48	0.55
DL-16	272.4	8	128.3	3.11	Moderately soluble	High	-4.64	0.55

Supplemental Table 2. Sequences of oligonucleotides used for PCR.

Gene	Primetime Probe (5'-3')	Forward (5'-3')	Reverse (5'-3')
AFP	TTGGAGAAGTACGGACATTCA GACTGC	CTGCAATTGAGAAACCCACTG	TTCCCTCTTCSCTTTGGCTG
ALB	TGCCTGCTGACTTGCCTTCATT AGCT	AAATCCCACTGCATTGCCGAAGTG	AGCAGCAGCACGACAGAGTAATCA
ASGR1	CGTGAAGCAGTTCGTGTCTGA CCT	TCCTTTCTGAGCCATTGCC	TGAAGTCGCTAGAGTCCCAG
APOB	CTGGATACCGTGTATGGAAACT GCTCC	CATTGCCCTTCCTCGTCTT	CCAGAGACAGAAGAAGCCAAG
HNF4a	CAAGAAATGCTTCCGGGCTGG C	TGGACAAAGACAAGAGGAACC	ATAGCTTGACCTTCGAGTGC
SLC10A1	AACCTCAGCATTGTGATGACCA CCT	TGTACAGGAGGAGAGGCATC	ACCTGTCCAATGTCTTCAGTC

Supplementary Materials

1. Supplemental Methods

Synthesis of 1,3,5-triazine-2,4-dithiol compounds and derivatives.

<u>Overview</u>

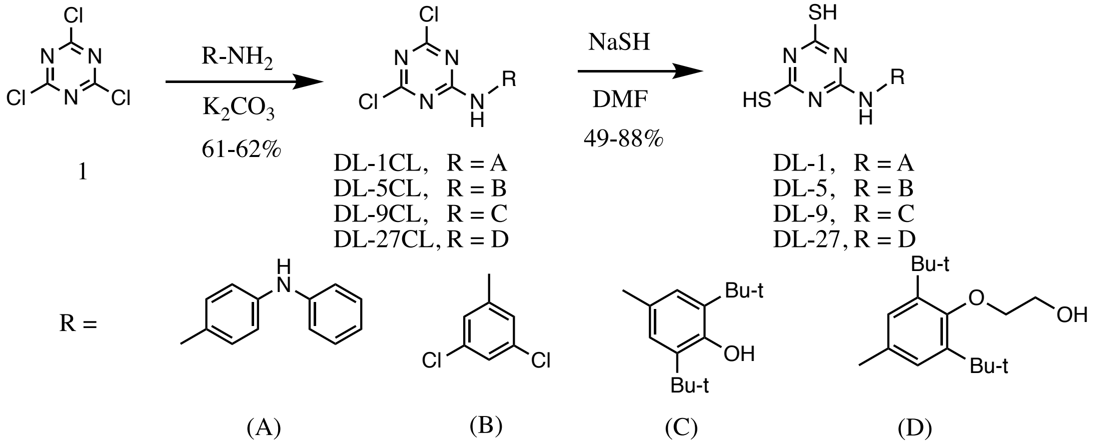

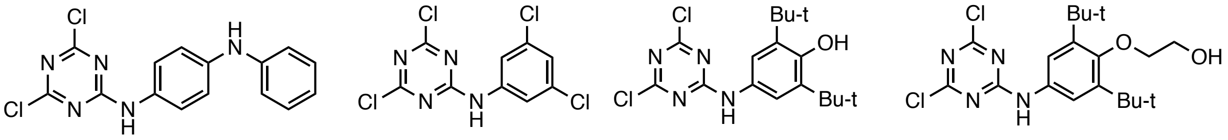

DL-1CL DL-5CL DL-9CL DL-27CL

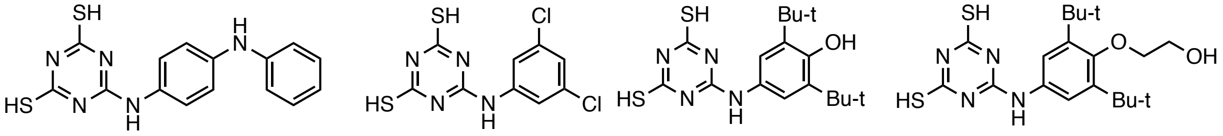

DL-1 DL-5 DL-9 DL-27

<u>General</u>

All chemicals were purchased from various commercial sources and used without further purification. Flash chromatography was performed with silica gel (70–230 mesh from Sorbent Technologies) and monitored by thin layer chromatography (TLC) with silica gel plates (Merck, Kieselgel 60 F_{254}). The ^{1}H spectra were recorded on a 400 MHz Bruker instrument. The spectra were recorded in hexadeuterioacetone (CD_3COCD_3) or hexadeuteriodimethylsulfoxide (DMSO-d_6). Chemical shifts of protons are given in parts per million with the solvent as internal standard. ESI-TOF high accuracy spectra were recorded on an Agilent 6230 TOF LC/MS system.

Synthesis of DL-1

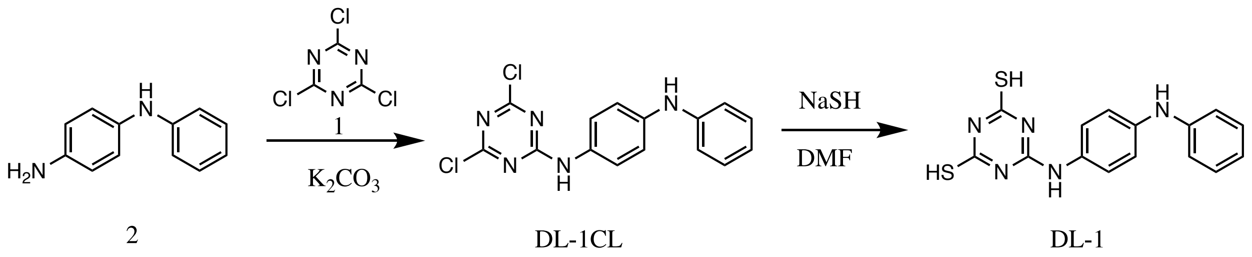

Synthesis of compound DL-1CL[1]

To a solution of cyanuric chloride (1, 2.31 g, 12.5 mmol) in tetrahydrofuran (THF, 12 mL), was added anhydrous potassium carbonate (3.46 g, 25 mmol) under nitrogen, while stirring with a magnetic stirrer. To this suspension, N-phenyl-1,4-benzenediamine (2, 2.31 g, 2.5 mmol) solution in tetrahydrofuran (13 ml) was then added dropwise through an addition funnel within 20 min. The resulted mixture was stirred overnight at room temperature, and then filtered through Celite. The mother liquid was concentrated under vacuum, and the residue was submitted to purification with column chromatography using silica gel and solvent system from hexanes to 70/30 (v/v, volume/volume) dichloromethane/hexanes, affording compound DL-1CL as an off-gray solid (2.55 g, 61%). ^{1}H NMR (DMSO-d^6, ppm) δ 6.83 (t, 1H, J = 8 Hz, Ar-H); 7.06-7.11 (m, 4H, Ar-H); 7.21-7.26 (m, 2H, Ar-H); 7.42 (d, 2 H, J = 8 Hz, Ar-H), 8.22 (s, 1 H, N-H); 10.97 (s, 1 H, N-H). ESI-TOF high accurate mass (M + H$^+$) for $C_{15}H_{12}N_5Cl_2$, calculated, 332.046; found, 332.047.

Synthesis of DL-1[1]

A solution of compound DL-1CL (2.83 g, 8.5 mmol) in dimethylformamide (DMF, 10 mL) was prepared first under nitrogen while stirring. This solution was then cooled to 0°C on ice-water bath and water (1.36 ml) was added. To this solution, a solution of sodium hydrosulfide (NaSH, 2.125 g, 25.5 mmol) in water (5.0 mL) was added within 2 h. After the completion of the addition, the resulted mixture was stirred at 0°C for 1 h, and 90°C for 1 h. After cooling at room temperature, the mixture was poured on to water (70 mL) and acidified with 3 N HCl to pH 3. The resulting precipitate was filtered, washed with water and recrystallized from DMF (25 mL) by heating, affording DMF containing solid (3.62 g). This solid was dissolved in DMSO (9 mL) by heating. The resulting solution was added onto water (400 mL) while stirring. The resulting precipitate was filtered, washed with water and dried in the air, affording DMF free DL-1 as a light yellow solid (2.44 g, 88%). ^{1}H NMR (DMSO-d^6, ppm) δ 6.83 (t, 1H, J = 8 Hz, Ar-H); 7.06-7.11 (m, 4H, Ar-H); 7.21-7.26 (m, 2H, Ar-H); 7.41 (d, 2 H, J = 8 Hz, Ar-H), 8.24 (s, 1 H, N-H); 9.08 (s, 1 H, N-H); 12.03 (s, 1 H, S-H), 13.06 (s, 1 H, S-H). ESI-TOF high accurate mass (M + H$^+$) for $C_{15}H_{14}N_5S_2$ (M +H$^+$), calculated: 328.069; found: 328.069.

Synthesis of DL-5

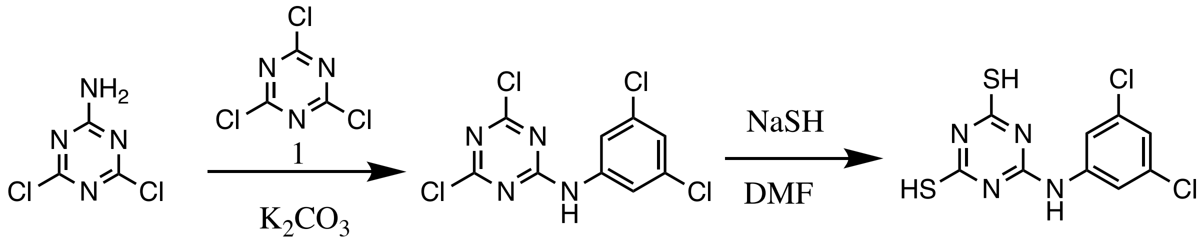

3 DL-5CL DL-5

Synthesis of compound DL-5CL

To a solution of cyanuric chloride (1, 1.84 g, 10.0 mmol) in THF (10 mL), was added anhydrous potassium carbonate (2.76 g, 20 mmol) under nitrogen, while stirring with a magnetic stirrer. To this suspension, 3,5-dichloroaniline (3, 1.62 g, 10.0 mmol) solution in tetrahydrofuran (10 ml) was then added dropwise through an addition funnel within 20 min. The resulted mixture was stirred overnight at room temperature, and then filtered through Celite. The mother liquid was concentrated under vacuum, and the residue was submitted to purification with column chromatography using silica gel and solvent system from hexanes to 50/50 (v/v, volume/volume) dichloromethane/hexanes, affording compound DL-5CL as a white solid (1.93 g, 62%). ^{1}H NMR (DMSO-d^6, ppm) δ 7.42 (t, 1 H, J = 4 Hz, Ar-H); 7.72 (d, 2 H, J = 4 Hz, Ar-H), 11.43 (s, 1 H, N-H). ESI-TOF high accurate mass (M + H^+) for $C_9H_5N_4Cl_4$, calculated, 308.926; found, 308.926.

Synthesis of DL-5

A solution of compound DL-5CL (1.55 g, 5.0 mmol) in DMF (14 mL) was prepared first under nitrogen while stirring. This solution was then cooled to 0°C on ice-water bath and water (0.8 mL) was added. To this solution, a solution of sodium hydrosulfide (1.25 g, 15.0 mmol) in water (2.5 mL) was added within 2 h. After the completion of the addition, the resulted mixture was stirred at 0°C for 1 h, and 90°C for 1 h. After cooling, the mixture was poured on to water (70 mL) and acidified with 3 N HCl to pH 3. The resulting precipitate was filtered, washed with water and recrystallized from DMF (20 mL) by heating, affording DMF containing solid (1.38 g). This solid was dissolved in DMSO (16 mL) by heating. The resulting solution was added onto water (320 mL) while stirring. The resulting precipitate was filtered, washed with water and dried in the air, affording DMF free DL-5 as a white solid (0.86 g, 53%). ^{1}H NMR (DMSO-d^6, ppm) δ 7.41 (t, 1 H, J = 4 Hz, Ar-H); 7.69 (s, 2 H, Ar-H), 9.43 (s, 1 H, N-H); 13.21 (s, 1 H, S-H). ESI-TOF high accurate mass (M - H^+) for $C_9H_5N_4Cl_2S_2$, calculated: 302.933; found: 302.926.

Synthesis of DL-9

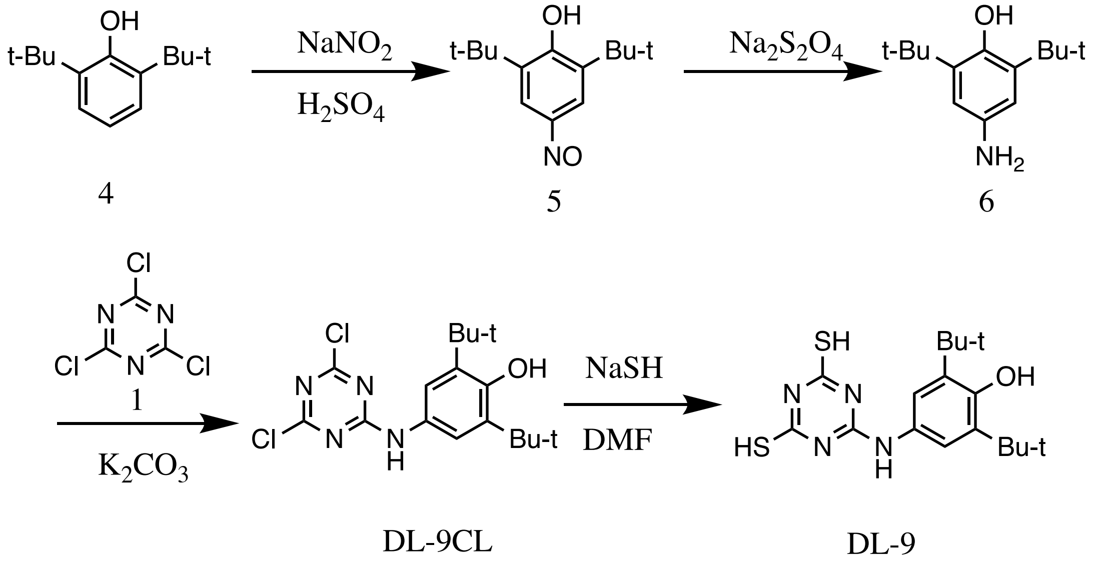

Synthesis of compound 5[2]

To a solution of 2,6-di-tert-butylphenol (4, 46.7 g, 227 mmol) in ethanol (200 mL), was added sulfuric acid (98%, 6.65 mL) and then a solution of sodium nitrite (16.15 g, 234 mmol) in water (50 mL) dropwise through an addition funnel at 0°C using a salt-ice water bath. During the reaction and when the reaction mixture was difficult to stir by a magnetic stirrer, manual shaking was applied. After the completion of the addition, the reaction mixture was sat for 75 min below 10°C, filtered, and washed with water and then dichloromethane, affording pure compound 5 as a white solid (36.6 g, 69%) based on thin layer chromatography. ^{1}H NMR (DMSO-d^6, ppm) δ 1.25 (s, 18 H, C-H); 6.99 (d, 1 H, J = 4 Hz, Ar-H); 7.49 (d, 1 H, J = 4 Hz, Ar-H); 13.24 (s, 1 H, O-H).

Synthesis of compounds 6 and DL-9CL

Compound 5 (2.35 g, 10.0 mmol) was dissolved in aqueous solution of sodium hydroxide (5%, 25 mL). The solution was heated to 50-60°C. To the hot solution, was added sodium hydrosulfite (4.5 g) by one portion. After the reaction mixture was maintained at this temperature for 1 h, 20 ml of water was added. The resulted mixture was then cooled to room temperature. The reaction mixture was filtered using a filter funnel and washed with water. The solid on the filter funnel was dissolved into dichloromethane. The organic layer was separated from aqueous layer using a separation funnel and dried over anhydrous sodium sulfate. The organic layer was then concentrated in vacuum, affording compound 6, which is preserved in nitrogen atmosphere and used in next step without further purification.

To a solution of cyanuric chloride (2, 1.84 g, 10.0 mmol) in tetrahydrofuran (10 mL), was added anhydrous potassium carbonate (2.76 g, 20 mmol) under nitrogen, while stirring with a magnetic stirrer. To this suspension, a solution of compound 6 in tetrahydrofuran (10 ml) was then added dropwise through an addition funnel within 20 min. The resulted mixture was stirred overnight at room temperature, and then filtered through Celite. The mother liquid was concentrated under vacuum, and the residue was submitted to purification with column chromatography using silica gel and solvent system from hexanes to 40/60 (v/v, volume/volume)

dichloromethane/hexanes, affording compound DL-9CL as a white solid (2.42 g, 66% for two steps).^{1}H NMR (DMSO-d^6, ppm) δ 1.38 (s, 18 H, C-H); 7.00 (s, 1 H, N-H); 7.42 (s, 2 H, Ar-H); 10.82 (s, 1 H, O-H). ESI-TOF high accurate mass (M + H^+) for $C_{17}H_{23}N_4OCl_2$, calculated, 369.124; found, 369.126.

Synthesis of DL-9

A solution of compound DL-9CL (1.845 g, 5.0 mmol) in dimethylformamide (6 mL) was prepared first under nitrogen while stirring. This solution was then cooled to 0°C on ice-water bath and water (0.8 mL) was added. To this solution, a solution of sodium hydrosulfide (1.25 g, 15.0 mmol) in water (3.0 mL) was added within 2 h. After the completion of the addition, the resulted mixture was stirred at 0°C for 1 h, and 90°C for 1 h. After cooling, the mixture was poured on to water (40 mL) and acidified with 3 N HCl to pH 3. The resulting precipitate was filtered, washed with water and recrystallized from DMF (12 mL) by heating, affording DMF containing solid (1.23 g). This solid was dissolved in DMSO (4.5 mL) by heating. The resulting solution was added onto water (200 mL) while stirring. The resulting precipitate was filtered, washed with water and dried in the air, affording DMF free DL-9 as a white solid (0.90 g, 49%). ^{1}H NMR (DMSO-d^6, ppm) δ 1.38 (s, 18 H, C-H); 7.04 (s, 1 H, N-H); 7.30 (s, 2 H, Ar-H); 8.99 (s, 1 H, O-H); 12.03 (s, 1 H, S-H); 13.02 (s, 1 H, S-H). ESI-TOF high accurate mass (M + H^+) for $C_{17}H_{25}N_4OS_2$ (M +H^+), calculated: 365.146; found: 365.147.

Synthesis of DL-27

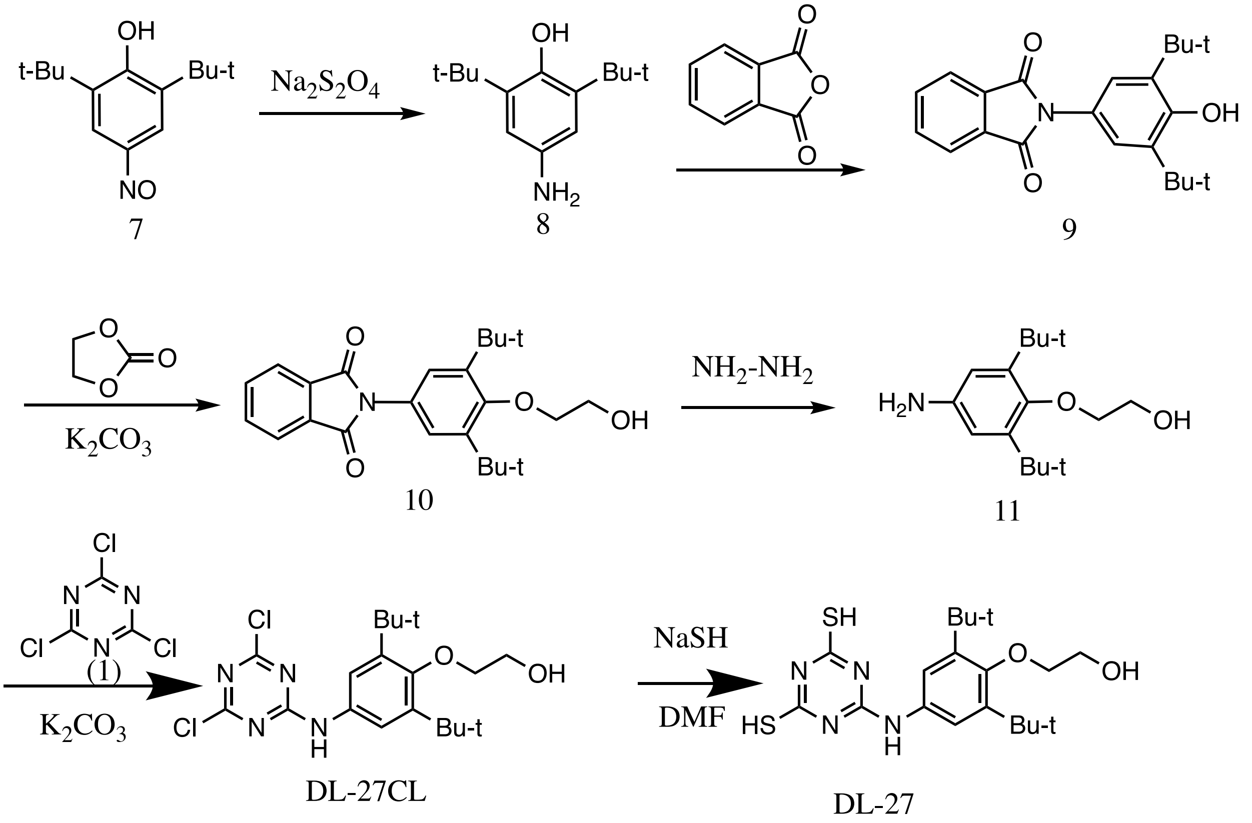

Synthesis of compounds 8 and 9

Compound 7 (27.6 g, 117 mmol) was dissolved in aqueous solution of sodium hydroxide (5%, 300 mL). The solution was heated to 50-60°C. To the hot solution, was added sodium hydrosulfite (52.85 g) by one portion. When two thirds of sodium hydrosulfite had been added, the mixture was difficult to stir with a magnetic stirrer. Manual shaking was then applied. After the reaction mixture was maintained between 50 and 75°C for 1 h, 250 ml of water was added. The resulted mixture was then cooled to room temperature. The reaction mixture was filtered using a filter funnel and washed with water. The solid on the filter funnel was dissolved with dichloromethane. The organic layer was separated from aqueous layer using a separation funnel and dried over anhydrous sodium sulfate. The organic layer was then concentrated in vacuum, affording compound 8, which is preserved in nitrogen atmosphere and used in next step without further purification.

To a solution of compound 8 in tetrahydrofuran (THF, 300 mL), was added phthalic anhydride (17.2 g, 117 mmol) under nitrogen. After the resulted mixture was refluxed on an oil bath for 24 h, concentrated hydrochloric acid (36.5%, 1.5 mL) was added. The reaction mixture was further refluxed for 17 h. The mixture was then cooled to room temperature and concentrated in vacuum. The residue was dissolved in a mixed solvent of acetone (200 mL) with methanol (200 mL). To this mixture, sodium bicarbonate (5%, 130 mL) was added. The resulted mixture was stirred at 60°C for 1 h to remove residual phthalic anhydride. After the reaction mixture was cooled to room temperature, it was concentrated in vacuum and extracted with ethyl acetate (3 x 150 mL). The organic layer was dried over anhydrous magnesium sulfate and concentrated in vacuum to a moment, when significant crystallization took place. The crystals were separated by filtration and washed with a mixed solvent of acetone with hexanes (1/3, v/v). The mother liquid was further concentrated, and crystals were filtered and washed with the mixed solvent. The process was repeated for one more time, affording compound 9 as a white solid (total 21.5 g, 52%). ^{1}H NMR (acetone-d^6, ppm) δ 1.48 (s, 18 H, C-H); 6.34 (s, 1 H, O-H); 7.29 (s, 2 H, C-H); 7.89-7.96 (m, 4 H, Ar-H). ESI-TOF high accurate mass (M + H$^+$) for $C_{22}H_{26}NO_3$, calculated, 352.191; found, 352.191.

Synthesis of compound 10[3]

To a mixture of compound 9 (0.64 g, 1.82 mmol), ethylene carbonate (0.32 g, 3.64 mmol) and DMF (2.5 mL), was added potassium bicarbonate (0.404 g, 2.92 mmol). The resulted suspension was refluxed under nitrogen atmosphere for 2 h. After the mixture was cooled to room temperature, water (20 mL), acetone (20 mL) and sodium hydroxide (5%, 20 mL) were then added. This mixture was stirred for 5 minutes at room temperature, concentrated in vacuum and extracted with ethyl acetate (2 x 30 mL). The organic layer was dried over magnesium sulfate and then concentrated in vacuum. The residue was submitted to purification with column chromatography using silica gel and solvent system from hexanes to 20/80 (v/v) acetone/hexanes, affording compound 10 as a white solid (0.12 g, 17%). ^{1}H NMR (acetone-d^6, ppm) δ 1.48 (s, 18 H, C-H); 3.96-4.02 (m, 4 H, C-H); 7.43 (s, 2 H, C-H); 7.91-7.97 (m, 4 H, Ar-H).

Synthesis of compounds 11 and DL-27CL

To a solution of compound 10 (0.24 g, 0.61 mmol) in methanol (15 mL) was added hydrazine (65%, 1.19 g). The resulted mixture was heated at 65°C for 2 h. To the mixture, anhydrous ethanol (40 mL) was added. The resulted mixture was then concentrated in vacuum to almost dry. To the residue, more anhydrous ethanol (40 mL) was added. The resulted suspension was concentrated in vacuum again to almost dry. To the residue, dichloromethane (50 mL) was added. The suspension was filtered and washed with dichloromethane. The mother liquid was collected, dried over magnesium sulfate and concentrated in vacuum, affording compound 11, which is being used in next step without further purification.

To a solution of cyanuric chloride (0.132 g, 0.71 mmol) in tetrahydrofuran (5 mL), was added anhydrous potassium carbonate (0.195 g, 1.42 mmol) under nitrogen, while stirring with a magnetic stirrer. To this suspension, a solution of compound 11 in tetrahydrofuran (10 ml) was then added dropwise through an addition funnel within 20 min. The resulted mixture was stirred overnight at room temperature, and then filtered through Celite. The mother liquid was concentrated under vacuum, and the residue was submitted to purification with column chromatography using silica gel and solvent system from hexanes to 20/80 (v/v) ethyl acetate/hexanes, affording compound DL-27CL as a white solid (0.119 g, 47% for two steps). ESI-TOF high accurate mass (M + H^+) for $C_{19}H_{27}N_4O_2Cl_2$ (M + H^+), calculated: 413.151; found: 413.150.

Synthesis of DL-27

A solution of compound DL-27CL (0.119 g, 0.288 mmol) in dimethylformamide (3.0 mL) was prepared first under nitrogen while stirring. This solution was then cooled to 0°C on ice-water bath and water (0.3 ml) was added. To this solution, a solution of sodium hydrosulfide (0.26 g) in water (0.6 mL) was added within 2.5 min. After the completion of the addition, the resulted mixture was stirred at 0°C for 1 h, and 60°C for 1 h. After cooling, the mixture was poured on to water (30 mL) and acidified with 3 N HCl to pH 3. The resulting precipitate was filtered, washed with water and dried in the air, affording compound DL-27 as a white solid (90.7 mg, 77%). ^{1}H NMR (DMSO-d^6, ppm) δ 1.39 (s, 18 H, C-H); 3.73-3.79 (m, 4 H, C-H); 7.46 (s, 2 H, Ar-H); 7.46 (s, 1 H, N-H); 9.12 (s, 1 H, N-H); 13.10 (s, 1 H, S-H). ESI-TOF high accurate mass (M + H^+) for $C_{19}H_{29}N_4O_2S_2$ (M +H^+), calculated: 409.173; found: 409.174.

References

1. Lee, H-J; Sarwade, B. D.; Park, J.; Kim, E. Synthesis of new photopolymeric methacrylate thioester with s-triazine ring for holographic recording. *Optical Materials* **2007**, *30(4)*, 637-644.

2. Um, S.-I.; Lee, J.-K.; Kang, Y.; Baek, D.-J. The synthesis and properties of triazine-stilbene fluorescent brighteners containing the phenolic antioxidant. *Dyes and Pigments* **2004**, *64(2)*, 93-99.

3. Pallavicini, M.; Fumagalli, L.; Gobbi, M.; Bolchi, C.; Colleoni, S.; Moroni, B.; Pedretti, A.; Rusconi, C.; Vistoli, G.; Valoti, E. QSAR study for a novel series of ortho disubstituted phenoxy analogues of α1-adrenoceptor antagonist WB4101. European Journal of Medicinal Chemistry **2006**, *41(9)*, 1025-1040.
