# Supplementary material for: A human iPSC-derived hepatocyte screen identifies compounds that inhibit production of Apolipoprotein B
Source: Commun Biol. 2023 Apr 24;6:452. doi: 10.1038/s42003-023-04739-9 (PMC10125972; doi:10.1038/s42003-023-04739-9)
Supplement: Supplementary file 2 — Description of Additional Supplementary Files [file 42003_2023_4739_MOESM2_ESM.pdf]

Description of Additional Supplementary Files

File Name: Supplementary Data

Description: Compressed file containing all of the source data used to generate the graphs presented in the main figures in the paper along with original blots for Figure 3.
