# Supplementary figures and images for: A human iPSC-derived hepatocyte screen identifies compounds that inhibit production of Apolipoprotein B
Source: Commun Biol. 2023 Apr 24;6:452. doi: 10.1038/s42003-023-04739-9 (PMC10125972; doi:10.1038/s42003-023-04739-9)

\* Precision Plus Protein™ All Blue  
 Prestained Protein Standards #1610373  
**kDa mark\***

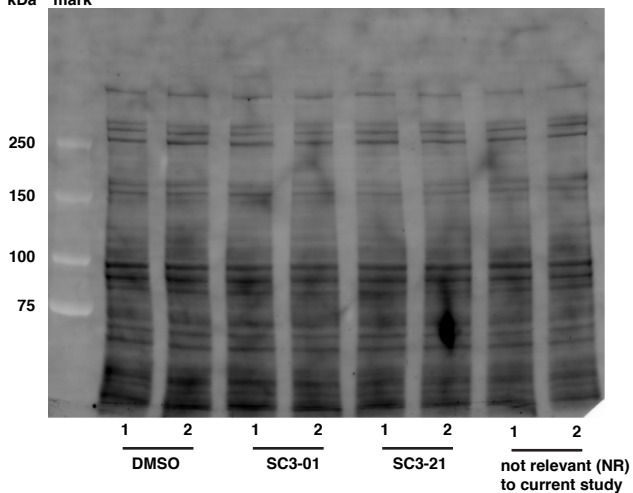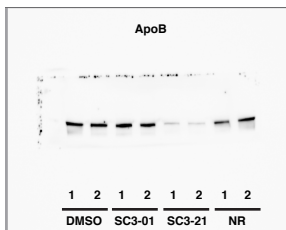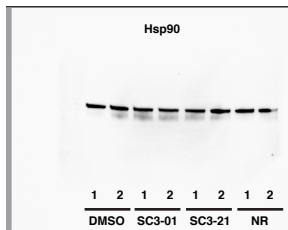

Supplement: Supplementary file 3 — Supplementary Data [file 42003_2023_4739_MOESM3_ESM.zip › Supplemental Data _ Main Figures/Figure 3H_Blot_SAD.pdf]
